# Prevalence and genetic diversity of respiratory syncytial viruses circulating in Bulgaria, 2023-2025

**DOI:** 10.3389/fcimb.2026.1844212

**Published:** 2026-07-24

**Authors:** Neli Korsun, Ivelina Trifonova, Yordanka Uzunova, Petar Velikov, Diyana Pavlova, Iliana Grigorova, Ivan Ivanov, Daniel Ivanov, Silviya Voleva, Iva Christova

**Affiliations:** 1 National Reference Laboratory “Influenza and ARD”, Department of Virology, National Center of Infectious and Parasitic Diseases, Sofia, Bulgaria; 2 Center of Competence “ImmunoPathogen”, Sofia, Bulgaria; 3 University Hospital “Lozenetz”, Sofia, Bulgaria; 4 Department for Infectious Diseases, Parasitology and Tropical Medicine, Medical University of Sofia, Sofia, Bulgaria; 5 Infectious Disease Hospital “Prof. Ivan Kirov”, Sofia, Bulgaria

**Keywords:** respiratory syncytial virus, acute respiratory infections, genetic diversity, monoclonal antibodies, whole-genome sequencing, three-dimensional protein model

## Abstract

**Background:**

Respiratory syncytial virus (RSV) is a major cause of acute respiratory infections (ARIs) with a considerable disease burden among children under 5 years of age. This study aimed to characterize the prevalence of RSV, genetic diversity of circulating strains, and amino acid variability in viral proteins, as well as identify mutations within the binding sites of monoclonal antibodies (mAbs) approved for RSV prophylaxis.

**Methods:**

Nasopharyngeal specimens from 5,170 patients of all ages with ARI were tested for 13 respiratory viruses using multiplex real-time PCR. Representative RSV-positive samples were subjected to whole-genome sequencing, followed by phylogenetic and amino acid sequence analyses.

**Results:**

RSV was positive in 303 (5.9%) patients, of whom 61 (20.1%) had co-infections with other respiratory viruses. Among children under 5 years of age, RSV was the most frequently detected pathogen in cases of bronchiolitis (30.6%) and pneumonia (12.6%). RSV-positive samples from the 2024-2025 season were genotyped, and predominance of RSV-A over RSV-B was observed. Phylogenetic analysis identified eight and seven genetic lineages within RSV-A and RSV-B, respectively, with A.D.1.11 and B.D.1.1 being the most prevalent. No substitutions were detected in the F protein antigenic sites of RSV-A (Ø, I-V), including the binding sites for nirsevimab (site Ø), palivizumab (site II), and clesrovimab (site IV). Conversely, RSV-B strains exhibited substitutions in antigenic sites Ø (n=3), I (n=2), and V (n=2).

**Conclusions:**

RSV is a leading cause of severe respiratory disease in early childhood and demonstrates substantial genetic diversity among circulating strains. The absence of resistance-associated substitutions in the F protein indicates that currently circulating RSV strains remain susceptible to approved mAbs.

## 1 Introduction

Respiratory syncytial virus (RSV) is a highly contagious respiratory pathogen that infects almost all children by age 2 and causes recurrent infections throughout life due to incomplete, short-lived immunity. It is a major cause of severe lower respiratory tract infections (LRTIs) in infants, young children, older adults, and immunocompromised individuals (Terstappen et al., 2024). This pathogen is responsible for approximately 33 million episodes of acute LRTIs worldwide, resulting in more than 3.6 million hospital admissions and 101,400 deaths annually among children aged 0 to 60 months (Li et al., 2022). RSV contributes significantly to pediatric hospitalizations, with acute RSV-associated bronchiolitis being the leading cause of hospital admissions in early childhood (Wildenbeest et al., 2023).

RSV is an enveloped virus belonging to the *Pneumoviridae* family, genus Orthopneumovirus (Lefkowitz et al., 2018). Its linear, single-stranded, negative-sense RNA genome encodes the NS1, NS2, N, P, M, SH, G, F, M2-1, M2-2, and L proteins (Collins and Karron, 2013). The surface glycoproteins G and F mediate viral entry and represent the primary targets of host immune responses. The G protein facilitates viral attachment to host cells and exhibits the highest sequence variability among RSV proteins, contributing to extensive genetic diversity. Its ectodomain contains two hypervariable regions (HVR1 and HVR2). In contrast, the F protein is more conserved and mediates the fusion of the viral envelope with the host cell membrane, wherein it undergoes a conformational transition from a metastable pre-fusion state to a highly stable post-fusion state. The pre-fusion conformation contains six key epitopes (Ø and I–V) and elicits substantially higher neutralizing antibody responses compared with that of the post-fusion form. Notably, antigenic sites Ø and V are unique to the pre-fusion conformation, whereas sites I–IV are present in both conformations (Bizot et al., 2025; Li et al., 2025). The stabilized pre-fusion structure of the F protein is a central focus of current next-generation vaccines and monoclonal antibody-based interventions since it is the primary target of neutralizing antibodies.

RSV comprises a single serotype and is classified into RSV-A and RSV-B antigenic groups, which co-circulate during epidemic seasons and often display alternating patterns of predominance (Salimi et al., 2021). Historically, RSV classification has been based on genetic variability within the HVR2 of the G gene. To date, more than 20 RSV-A and 30 RSV-B genotypes have been described across diverse geographical regions (Gaymard et al., 2018). In 2020, Goya et al. proposed an alternative framework incorporating genotypes, subgenotypes, and lineages based on sequence variability within the G gene ectodomain, which comprises approximately 550–600 bp. This approach consolidated previously defined genotypes into three major groups for RSV-A (GA1–GA3) and seven for RSV-B (GB1–GB7) (Goya et al., 2020). More recently, a standardized phylogenetic classification proposed in 2024 by the RSV Genotyping Consensus Consortium (RGCC) is based on whole-genome sequencing (WGS) and is implemented within the Nextclade analytical framework. This novel system defines 24 and 16 major lineages for RSV-A and RSV-B, respectively, each representing a monophyletic cluster of at least 10 sequences and characterized by more than five amino acid substitutions relative to the parental lineage (Goya et al., 2024). Multiple RSV lineages may co-circulate within a single epidemic season and population, with predominant lineages undergoing periodic replacement on a timescale of one to two years, although this may differ by region. Pronounced geographic and temporal variability in lineage distribution has been consistently documented (Wei et al., 2024; Martinez-Marrero et al., 2025; Franco et al., 2025). Therefore, continuous molecular surveillance of circulating RSV genotypes and lineages is essential for understanding RSV epidemiology and disease burden, enabling early detection of emerging variants and informing the development and optimization of preventive and therapeutic strategies.

For more than two decades, the only available preventive option against RSV infection has been the monoclonal antibody (mAb) palivizumab (Synagis), administered monthly during the RSV season and restricted to infants at high risk of severe disease. However, recent years have seen substantial advances in both active and passive immunization strategies. Three vaccines—Arexvy (GSK), Abrysvo (Pfizer), and mResvia (Moderna), and two long-acting mAbs— nirsevimab (Beyfortus, AstraZeneca/Sanofi) and clesrovimab (Еnflonsia, MSD) — all targeting RSV F protein, have been approved (Yu et al., 2025). All three vaccines are indicated for adults aged ≥ 60 years, while Abrysvo is also approved for maternal immunization to prevent RSV-associated LRTIs in infants during the first six months of life. Nirsevimab and clesrovimab demonstrate improved neutralizing potency compared with that of palivizumab and provide single-dose protection throughout the RSV season in infants entering their first RSV season (Bizot et al, 2025; Terstappen et al, 2024). However, continuous genomic surveillance of circulating RSV strains remains essential for the early detection of genetic variants that may escape vaccine-induced immunity or develop resistance to mAbs.

RSV is not a notifiable infectious disease in Bulgaria. However, integrated surveillance of respiratory viruses—including influenza, SARS-CoV-2, and RSV—is conducted in accordance with the recommendations of the European Centre for Disease Prevention and Control (ECDC). Thus, this study aimed to characterize the circulation patterns of RSV and other respiratory viruses during the first two post-pandemic seasons, describe the epidemiological and clinical features of RSV infection, and assess amino acid variation across the viral proteome, focusing on antigenic sites targeted by approved mAbs.

## 2 Materials and methods

### 2.1 Patients and specimen collection

This prospective study was conducted from October 2023 to May 2025, encompassing two respiratory virus seasons (2023–2024 and 2024–2025), defined from week 40 of the preceding year to week 20 of the following year. A total of 5,170 patients with medically attended ARIs from different regions of the country were enrolled through the National Influenza Surveillance Program. The ECDC case definition of ARI (https://ecdc.europa.eu/en/infectious-diseases-public-health/surveillance-and-disease-data/eu-case-definitions) and the WHO case definition of severe acute respiratory infections (SARIs) (WHO, 2013) were used to enroll participants in the study. The study population included patients of all ages: 1,882 in the first season, 2,981 in the second, and 307 during the inter-seasonal period (June to September 2024).

Nasopharyngeal and oropharyngeal swabs were collected from outpatients during medical consultations or from hospitalized patients within the first 24 h of admission. Swabs were placed in 2 mL of viral transport medium and sent to the National Reference Laboratory “Influenza and ARD” under refrigerated conditions using ice packs. Specimens were processed immediately upon receipt, and aliquots of primary samples were stored at -80 °C.

### 2.2 Molecular detection of respiratory viruses

Viral nucleic acids were extracted from 400 μL of collected specimens and eluted to a final volume of 100 μL using the commercial ExiPrep Dx Viral DNA/RNA kit (Bioneer, Republic of Korea) according to the manufacturer’s instructions. Detection of influenza A and B viruses and SARS-CoV-2 was performed using the FluSC2 Multiplex Real-Time RT-PCR Kit (International Reagent Resource [IRR], USA) in combination with the Applied Biosystems TaqPath™ 1-Step Multiplex Master Mix (Thermo Fisher Scientific), as previously described (Shu et al., 2021). Influenza-positive samples were further subtyped using the SuperScript III Platinum One-Step qRT-PCR kit (Invitrogen, Thermo Fisher Scientific, Waltham, MA, USA), and virus-specific primer/probe sets provided by IRR. Amplification was conducted according to the protocol recommended by the Centers for Disease Control and Prevention (CDC, Atlanta, GA, USA) (Shu et al., 2011).

A laboratory-developed multiplex real-time PCR assay was used to detect eight common non-influenza respiratory viruses, namely RSV, human metapneumovirus (hMPV), parainfluenza virus (PIV) types 1–3, rhinovirus (RV), adenovirus (AdV), and bocavirus (BoV). Three reaction mixtures, containing combinations of primers and TaqMan probes, as previously described (Kodani et al., 2011) and labeled with different fluorescent dyes, were used along with appropriate positive and negative controls. Amplifications were performed on a CFX96 thermal cycler (Bio-Rad Laboratories, Inc., Singapore). A cycle threshold (Ct) value of ≤ 38 was considered indicative of a positive result.

Genotyping of RSV-positive samples from the 2024-2025 season was performed using multiplex real-time RT-PCR with two sets of oligonucleotides targeting the RSV F and N genes, as previously described (Zlateva et al., 2007).

### 2.3 RSV whole-genome sequencing

RSV-A and RSV-B-positive samples with high viral loads (Ct values ≤ 28) from different regions of the country were selected for WGS. Library preparation and target enrichment were performed using the Respiratory Virus Panel with Illumina RNA Prep with Enrichment (L) Tag (Illumina, San Diego, CA, USA), followed by sequencing on the Illumina MiSeq platform using the MiSeq Reagent Kit v3 (150-cycle configuration) (Illumina, San Diego, CA, USA), according to the manufacturer’s protocol (https://support-docs.illumina.com/LP/Illumina_RNA_Prep_Checklist/Content/LP/Illumina_RNA/RNA-Prep/Checklist.htm). Complete or near-complete RSV genomes were obtained from 19 RSV-A and 20 RSV-B-positive samples with coverage greater than 99%. All RSV sequences generated in this study have been deposited in the GISAID EpiRSV database under the accession numbers provided in the **Supplementary Appendix**.

### 2.4 Phylogenetic analysis

For phylogenetic analysis, RSV-A and RSV-B reference sequences were obtained from the RGCC GitHub page (https://github.com/rsv-lineages/lineage-designation-A) and (https://github.com/rsv-lineages/lineage-designation-B). The sequences analyzed were aligned with full-length reference sequences representing established lineages. Forty-five sequences of RSV strains recently circulating in diverse geographical regions, including Europe, North America, and Australia that were genetically closest to the Bulgarian sequences were retrieved from the GenBank database and also included. Sequence alignment and phylogenetic tree construction were performed using Geneious Prime v2020.1.2 (https://www.geneious.com/). Phylogenetic trees were inferred using the maximum-likelihood method under the Tamura–Nei nucleotide substitution model selected based on model testing performed in Geneious Prime v2020.1.2. In addition, the use of bootstrap analysis with 1,000 bootstrap replicates allowed statistical evaluation of the stability of the observed clusters and supports the reliability of the inferred phylogenetic relationships. Final trees were visualized, annotated, and color-coded using Interactive Tree of Life (iTOL) v6 (https://itol.embl.de/). Lineage assignment of RSV sequences was performed using Nextclade (https://clades.nextstrain.org) based on the classification framework proposed by Goya et al. (2024).

### 2.5 Amino acid sequence analysis and glycosylation prediction

The study sequences were aligned and compared with reference sequences EPI_ISL_412866 (hRSV/A/England/397/2017; lineage A.D.2.2.1) and EPI_ISL_1653999 (hRSV/B/Australia/VIC-RCH056/2019; lineage B.D.4.1.1), which were used as representatives for RSV-A and RSV-B, respectively. These reference sequences correspond to well-characterized, full-length representatives of the globally predominant ON1 and BA9 genotypes.

Potential N- and O-linked glycosylation sites were predicted using the NetNGlyc 1.0 (https://services.healthtech.dtu.dk/service.php?NetNGlyc-1.0) and NetOGlyc 4.0 (https://services.healthtech.dtu.dk/service.php?NetOGlyc-4.0) web servers, respectively, applying a prediction threshold of ≥ 0.5. N-linked glycosylation sites were identified based on the canonical sequon N–X–S/T, where X represents any amino acid except proline. Potential O-linked glycosylation sites were predicted at serine and threonine residues based on sequence context.

### 2.6 Three-dimensional models of the RSV-A and RSV-B G glycoprotein

Three-dimensional models of the RSV-A and RSV-B G glycoprotein were generated using the web-based AlphaFold Server (https://alphafoldserver.com/), a platform for protein structure prediction. The wild-type sequence (reference sequence) and a mutant (studied) sequence were used as inputs. Structural visualization and residue mapping were performed in UCSF ChimeraX (Pettersen et al., 2021). Mutation sites were highlighted and compared between the wild-type and mutant structures.

### 2.7 Statistics

Statistical analyses were performed using GraphPad Prism version 8.0 (GraphPad Software, San Diego, CA, USA) and SAS OnDemand for Academics version 9.4 (SAS Institute Inc., Cary, NC, USA). Categorical variables were summarized as counts and percentages and compared between groups using the chi-square (χ²) or Fisher’s exact test, as appropriate. Statistical significance was defined as a two-sided *p* value < 0.05. Continuous variables were presented as means or medians with interquartile ranges (IQRs), depending on data distribution. Logistic regression analysis was used to evaluate associations between demographic and clinical variables (age, sex, and clinical diagnosis) and RSV detection, with results reported as odds ratios (ORs) and 95% confidence intervals (CIs). Multivariable logistic regression models adjusted for age and sex were used to estimate adjusted odds ratios (aORs) for overall RSV infection, RSV-A, and RSV-B, as well as direct comparisons between RSV-A and RSV-B infections.

## 3 Results

### 3.1 Patient characteristics

Of the 5,170 patients enrolled, 1,726 (33.4%) were managed in primary healthcare settings, while 3,444 (66.6%) were hospitalized. Participants ranged in age from 10 days to 98 years, with a median age of 7 years (IQR 2-14 years). To assess age-specific infection patterns, the study population stratified into six age groups: 0-11 months (n=545, 10.5%), 12–35 months (n=882, 17.1%), 3-5 years (n=902, 17.4%), 6-17-years (n=1992, 38.5%), 18–64 years (n=476, 9.2%), and ≥ 65-years (n=426, 8.2%) Age data were missing for 17 (0.3%) patients. Among participants with available sex data, 2,676 (51.8%) and 2,476 (47.9%) were male and female, respectively, yielding a male-to-female ratio of 1.08.

### 3.2 Virus detection

**Figure 1:**
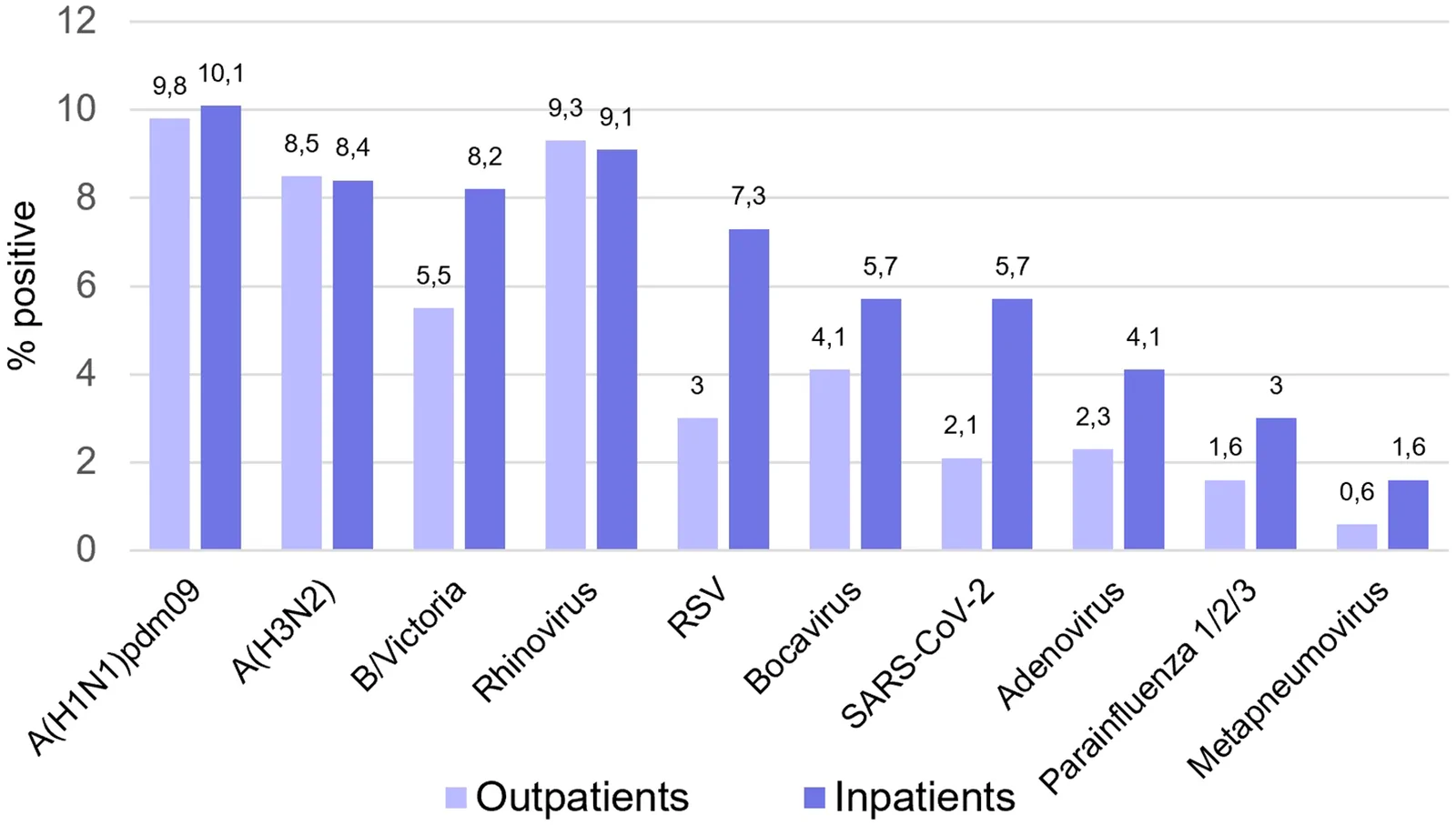
Proportions of detected respiratory viruses among outpatients and inpatients.

All 5,170 patient samples were screened for 13 respiratory viruses using real-time PCR. At least one respiratory virus was detected in 2,668 (51.6%) patients. Specifically, 896 (47.6%) patients tested positive during the 2023-2024 season, 1,641 (55%) patients during the 2024-2025 season, and 131 (42.7%) patients during the inter-seasonal period. A total of 747 (43.3%) and 1,921 (55.8%) virus-positive cases were confirmed in outpatients and inpatients, respectively (p < 0.0001). In the overall study population, 2,371 (45.9%) patients had single-virus infections, 273 (5.3%) had co-infections with two viruses, 23 (0.4%) had co-infections with three, and 1 (0.02%) had a co-infection with four viruses. SARS-CoV-2 was detected in 234 (4.5%) patients, while influenza viruses were identified in 1,329 (25.7%) patients. Influenza cases included A(H1N1)pdm09 (517 cases, 10,0% of all samples tested), A(H3N2) (436 cases, 8.4%), and B/Victoria lineage (376 cases, 7.3%). Non-influenza respiratory viruses were detected in 1,276 (24.7%) patients including RV in 475 (9.2%), RSV in 303 (5.9%), BoV in 266 (5.1%), AdV in 180 (3.5%), hMPV in 67 (1.3%), PIV-3 in 51 (1,0%) PIV-1 in 49 (0.9%), and PIV-2 in 30 (0.6%). RSV was the second most frequently detected seasonal non-influenza respiratory virus after RV. PIV types 1-3 and hMPV were detected at the lowest frequencies, each accounting for fewer than 70 cases. During the first and second seasons, RSV detection rates were 6.6% (125/1,882) and 6.0% (178/2,981), respectively. No RSV cases were detected during the inter-seasonal period. Among outpatients, the detection rate of RSV was 3.0% (51/1,726), whereas a significantly higher rate was observed in inpatients (7.3%, 252/3,444) (p < 0.0001). Other respiratory viruses were also significantly more frequently detected among the hospitalized patients than among the outpatients: SARS-CoV-2 (5.7% *vs* 2,1%; p = 0.0001), influenza B/Victoria (8.2% *vs* 5.5%; p = 0.0005), AdV (4.1% *vs* 2.3%; p = 0.0005), HMPV (1.6% *vs* 0.6%; p = 0.0025), PIV (3% *vs* 1.6%; p = 0.0058), and BoV (5.7% *vs* 4.1%; p = 0.0193) (**Figure 1**).

During the 2024–2025 season, all 178 RSV-positive samples were genotyped, and 91 (51.1%) of them were classified as RSV-A, 72 (40.4%) as RSV-B, and 4 (2.2%) as co-infections with both. Eleven (6.2%) samples were not subtyped due to low viral load. Among RSV-A-positive cases, 91.2% (83/91) and 8.8% (8/91) were identified in inpatients and outpatients, respectively. Similarly, among RSV-B-positive cases, 93.1% (67/72) and 6.9% (5/72) were identified in inpatients and outpatients, respectively. No statistically significant differences were observed in the distribution of RSV-A and RSV-B among inpatients (p = 0.2120) and outpatients (p = 0.5797).

**Figure 2:**
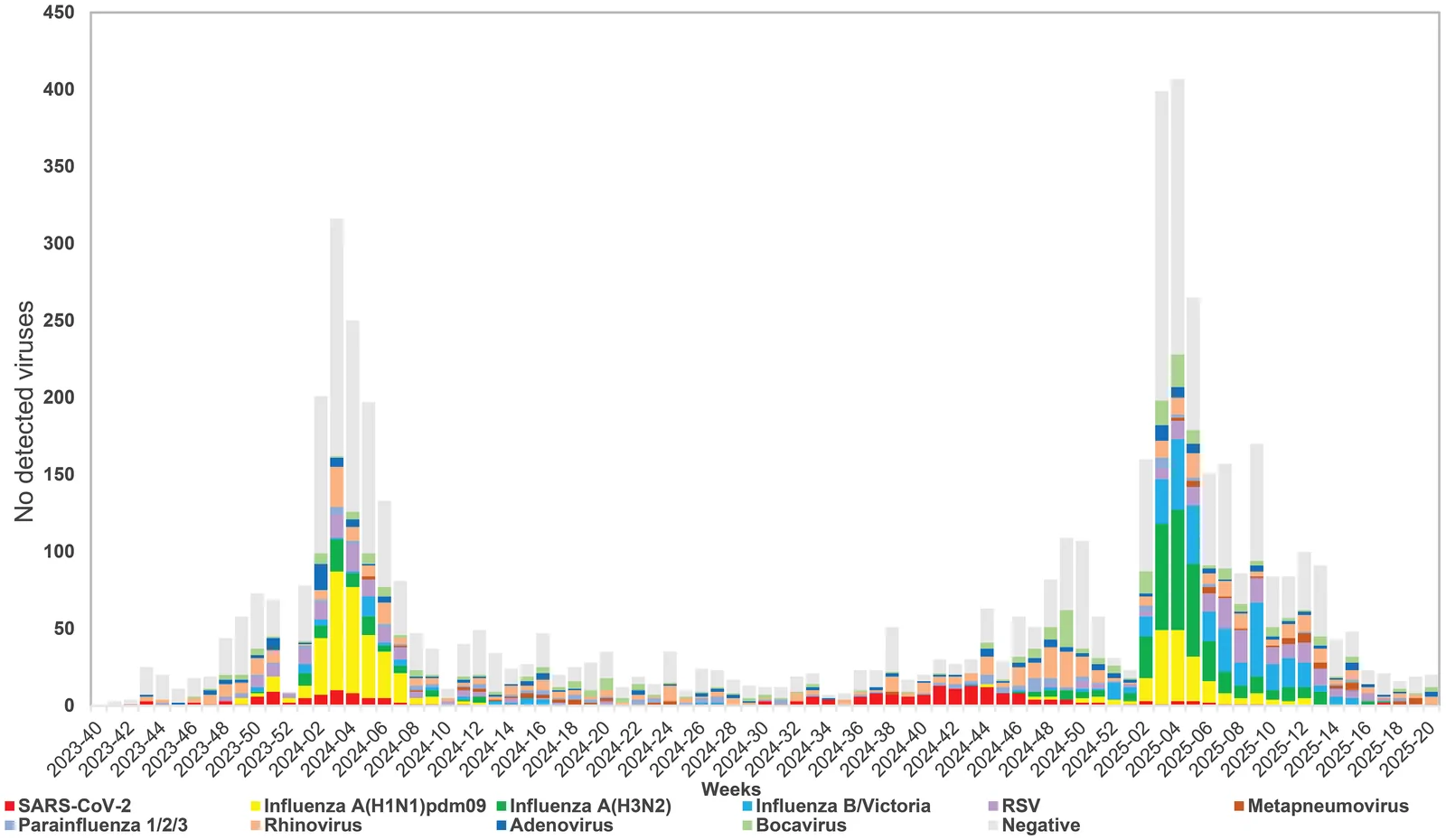
Weekly distribution of respiratory viruses detected among patients with ARI, 2023-2025.

During the 2023-2024 season, RSV circulation began in week 48 of 2023 and peaked in week 04 of 2024. In the next season, RSV circulation also began in week 48 (2024) but peaked later, in week 08 of 2025. In both seasons, RSV was detected between November and May. Across the study period, the highest RSV detection rate was observed in February 2025 (13.3%, 71 cases). RSV circulated alongside influenza viruses during the January-March period. SARS-CoV-2 activity was highest in the fall of 2024 and has decreased significantly since the start of influenza and RSV circulation (**Figure 2**).

### 3.3 Age and sex distribution

Age-dependent differences were observed in the detection rates of respiratory viruses. The proportions of virus-positive cases in the age groups 0–11 months, 12–35 months, 3-5 years, 6–17 years, 18–64 years, and ≥ 65 years were 60.4% (329/545), 64.1% (565/882), 57.4% (518/902), 45.3% (871/1,922), 41.6% (198/476), and 41.8% (178/426), respectively. The highest detection rate was observed in toddlers aged 12-35 months, followed by infants aged 0-11 months. However, the differences between these age groups were not statistically significant (p = 0.1765). In the 2024-2025 season, higher virus-positivity rates were observed compared to the previous season in the following age groups: 0-11 months (63.9% *vs* 54.1%; p = 0.0412), 3-5 years (63.5% *vs* 52,2%; p = 0.0009), and 6-17 years (50.8% *vs* 39.7%; p = 0.0001).

**Figure 3:**
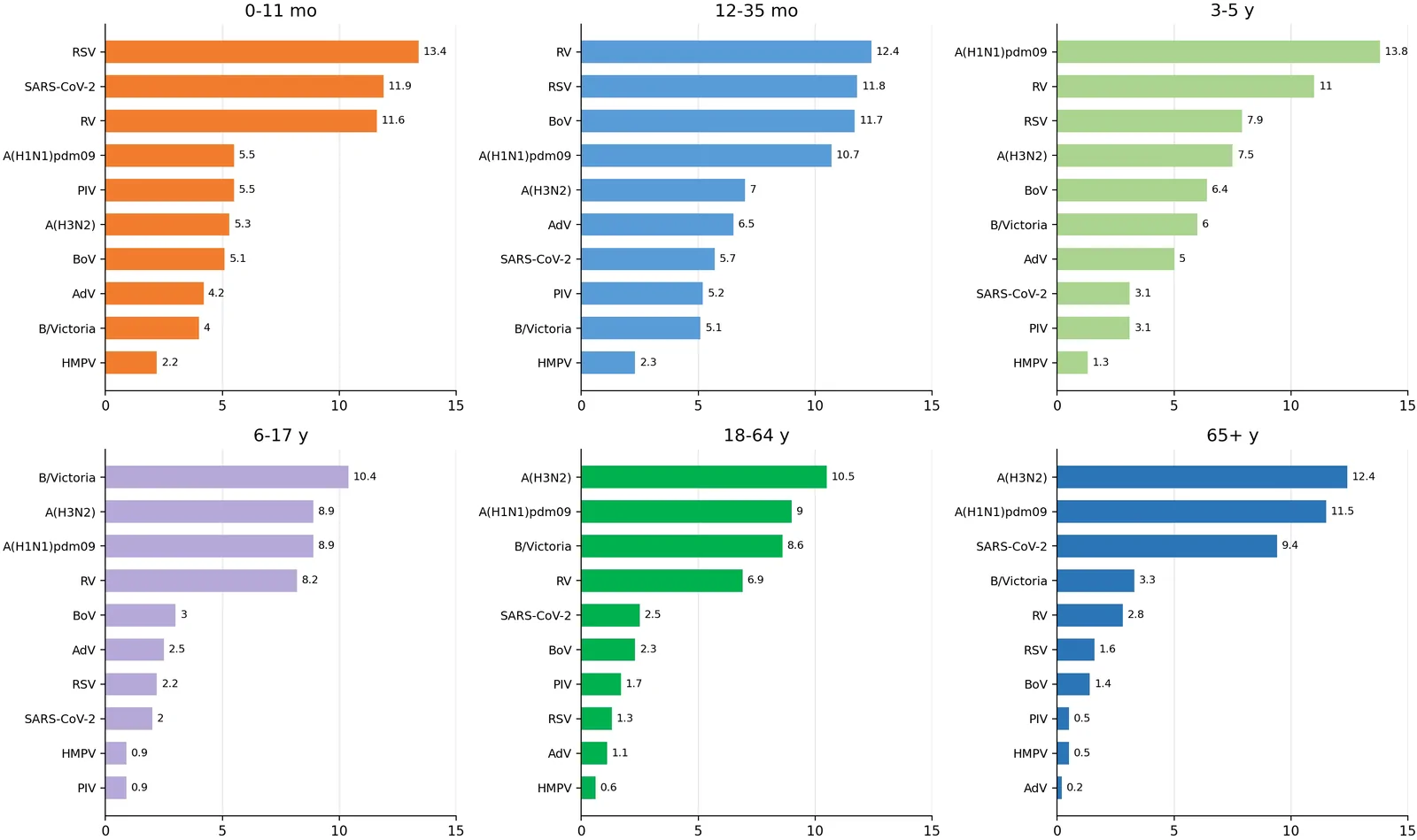
Proportions of patients from different age groups (0-11 months, 12-35 months, 3-5 years, 6-17 years, 18-64 years, and +65 years) infected by different respiratory viruses.

Patients with RSV infection ranged in age from 10 days to 81 years, with a mean age of 5.5 ± 13.53 years and a median age of 2 years (IQR 1–4 years). RSV-A-positive patients had a median age of 2 years (IQR 1–4 years), whereas RSV-B-positive patients had a median age of 1 year (IQR 3 months-3 years). The highest RSV detection rate was observed in the youngest age group (0–11 months) at 13.4%. Detection rates declined with increasing age, reaching 1.3% in the 18–64-year age group, with a slight increase to 1.6% in individuals aged ≥ 65 years (**Figure 3**). Among infants aged 0–6 months, the RSV-positivity rate was 13.2%, representing the highest rate among all respiratory viruses studied. A relatively high RSV detection rate was also observed in children aged 12–35 months (11.8%). Overall, most RSV infections occurred in children younger than 5 years, accounting for 81.8% (248/303) of all RSV-positive cases, corresponding to a detection rate of 10.6% (248/2,329) in this age group. Among RSV-positive children aged 0–5 years, 85.1% (211/248) were hospitalized. The detection rate of RSV among hospitalized children aged 0–5 years was 11.8% (211/1,788), higher than the 6.8% (37/541) among outpatients (p = 0.0008). In infants aged 0–11 months, these proportions were 14.0% (68/484) and 8.2% (5/61) among inpatients and outpatients, respectively (p = 0.2373). RSV was the most frequently detected respiratory virus among hospitalized infants aged 0–11 months. No significant differences were found in RSV-detection rates across age groups between the two seasons.

RSV infection was more frequent in males (7.0%, 187/2,676) than in females (4.6%, 115/2,476) (p = 0.0004). During the 2023-2024 season, RSV detection rates among males and females were 8% (82/1022) and 5% (43/856), respectively (p = 0.0093). During the next season, these proportions were 7% (106/1508) and 4.9% (72/1473), respectively (p = 0.0163). The male-to-female ratio among RSV-positive inpatients was 1.55 (152 males *vs.* 98 females) (p = 0.0025), and among outpatients it was 2.0 (34 males *vs.* 17 females) (p = 0.0463), both statistically significant. No significant sex-based differences in RSV prevalence were observed across individual age groups: 0-11 months (45/299 males and 28/242 females; p = 0.2567), 12-35 months (61/478 males and 43/403 females; p = 0.3478), 3-5 years (45/492 males and 25/407 females; p = 0.1046) 18-64 years (4/189 males and 2/287 females; p = 0.2207) and +65 years (1/164 males and 6/262 females; p = 0.2576). The exception was the age group 6–17–year age group, where RSV infection was significantly more frequent in males (30/1042) than in females (11/871) (p = 0.0169).

Univariate logistic regression analysis demonstrated that male sex was associated with an increased likelihood of RSV detection (**Supplementary Table 1**). Male patients had significantly higher odds of RSV detection compared with female patients ([OR] 1.55, 95% [CI] 1.22–1.98, p < 0.001). Age was also significantly associated with RSV detection. Compared with adults aged 18–64 years, infants aged 0–11 months had the highest odds of RSV detection (OR 11.36, 95% CI 4.88–26.45, p < 0.001), followed by children aged 12–35 months (OR 10.01, 95% CI 4.36–23.01, p < 0.001) and those aged 3–5 years (OR 6.47, 95% CI 2.79–15.01, p < 0.001). No significant differences were observed for children aged 6–17 years (OR 1.72, 95% CI 0.73–4.09) or older adults aged ≥ 65 years (OR 1.15, 95% CI 0.37–3.58).

No statistically significant differences were found in the distribution of RSV-A and RSV-B infections across various age groups, except for children aged 3-5 years (n = 488). In this age group, RSV-A was detected in 22 (4.5%) patients, while RSV-B was found in 10 (2%) patients (p = 0.0464). No differences between RSV-A and RSV-B were observed among male and female patients.

### 3.4 Viral detections in patients with different clinical diagnoses

Respiratory viral infections primarily present with upper respiratory tract symptoms but may also be associated with complications involving the lower respiratory tract, cardiovascular system, and central nervous system (CNS). In this study, we evaluated the contribution of RSV and other respiratory viruses to common clinical diagnoses, including bronchitis, bronchiolitis, pneumonia, and neurological manifestations (e.g., febrile seizures, cerebral edema, viral meningitis, and encephalopathy). Overall, 216 (4.2%) patients were diagnosed with bronchitis, 222 (4.3%) with bronchiolitis, 753 (14.6%) with pneumonia, and 107 (2.1%) with CNS manifestations. The detection rates of RSV among patients diagnosed with these conditions were 11.6% (25/216) for bronchitis, 30.6% (68/222) for bronchiolitis, 8.5% (64/753) for pneumonia, and 0.9% (1/107) for CNS manifestations. In contrast, influenza viruses were detected in 18.1% (39/216) of bronchitis cases, 12.2% (27/222) of bronchiolitis, 17.0% (128/753) of pneumonia, and 9.3% (10/107) of CNS. SARS-CoV-2 detection rates were lower across all conditions, with 0.9% (2/216) for bronchitis, 1.4% (3/222) for bronchiolitis, 1.5% (11/753) for pneumonia, and 0.9% (1/107) for CNS manifestations. RSV was the most frequently detected virus among patients with bronchiolitis (p < 0.0001). During the 2024-2025 season, the RSV detection rate in patients with bronchiolitis (38.3%; 49/128) was significantly higher than in the previous season (21.6%; 19/88) (p = 0.0112). However, in patients with pneumonia, the RSV detection rates in the two seasons were not significantly different: 10.1% (32/318) in the 2023-2024 season and 7.4% (32/432) in the 2024-2025 season (p = 0.2339). Among all children aged 0–5 years, 389 (16.7%) were diagnosed with pneumonia, of whom 49 (12.6%) and 47 (12.1%) were RSV- and influenza-positive, respectively. In infants aged 0–11 months with pneumonia (n=87), RSV was detected in 14 cases (16.1%) compared with 6 cases (6.9%) for influenza viruses. In infants aged 0–11 months with bronchiolitis (n=90), 26 (28.9%) were RSV-positive, whereas 10 (19.1%) were influenza-positive (p = 0.0047). During the 2024–2025 season, RSV-A accounted for 4.5% (7/155) of bronchitis cases, 17.2% (22/128) of bronchiolitis, and 5.8% (25/433) of pneumonia. Corresponding proportions for RSV-B were 5.2% (8/155), 19.5% (25/128), and 1.4% (6/433), respectively. RSV-A was more frequently detected in patients with pneumonia compared to RSV-B (p = 0.0007).

Clinical diagnosis was significantly associated with RSV detection (**Supplementary Table 1**). Compared with upper respiratory tract infection (133 RSV-positive/3391 cases tested), bronchiolitis showed the strongest association with RSV detection ([aOR] 5.31, 95% [CI] 3.69–7.63), followed by bronchitis (aOR 3.21, 95% CI 1.95–5.31) and pneumonia (aOR 2.01, 95% CI 1.46–2.75) (Supplementary Figure). In subtype-specific analyses, RSV-A detection was independently associated with bronchiolitis (aOR 11.54, 95% CI 6.55–20.30), bronchitis (aOR 3.42, 95% CI 1.49–7.84), and pneumonia (aOR 3.33, 95% CI 1.97–5.60). RSV-B detection was associated with bronchiolitis (aOR 12.01, 95% CI 6.83–21.14) and bronchitis (aOR 2.61, 95% CI 1.01–6.77), but not with pneumonia. By directly comparing RSV-A and RSV-B infections, pneumonia was more strongly associated with RSV-A compared with RSV-B (aOR 4.25, 95% CI 1.48–12.19).

### 3.5 Involvement of RSV in co-infections

In this study, more than one respiratory virus was detected in 297 (5.7%) patient’s samples. Among RSV-positive patients, a total of 61 (20.1%) were co-infected with other respiratory viruses: 24 (19.2%) during the first season and 37 (20.8%) during the second season. **Table 1** presents all co-infected samples with the corresponding Ct values for each detected pathogen. Among the samples with co-detection, only 3 (4.9%) had RSV Ct ≥ 35, potentially representing residual shedding rather than active co-infection.

**Table 1:** Number (%) of samples with RSV co-detections and corresponding Ct values for each detected pathogen.

Co-infection type	Frequency		RSV Ct		Co-pathogen Ct	
	n/303 (%)	n/61 (%)	Mean ± SD	Range	Mean ± SD	Range
RSV + Influenza A	13/303 (4.3%)	13/61 (21.3%)	26.7 ± 4.5	21.7–35.9	25.1 ± 3.3	19.9–31.5
RSV + Influenza B	7/303 (2.3%)	7/61 (11.5%)	28.9 ± 6.7	17.7–37.3	23.5 ± 5.7	15.8–32.4
RSV + SARS-CoV-2	7/303 (2.3%)	7/61 (11.5%)	24.1 ± 6.7	17.8–36.2	23.7 ± 3.4	17.8–28.0
RSV + BoV	19/303 (6.3%)	19/61 (31.1%)	25.7 ± 5.1	17.0–34.3	32.7 ± 4.6	20.1–38.0
RSV + RV	13/303 (4.3%)	13/61 (21.3%)	25.1 ± 3.8	20.2–32.8	29.9 ± 3.4	22.9–34.1
RSV + AdV	6/303 (2.0%)	6/61 (9.8%)	23.5 ± 6.6	18.4–26.0	30.0 ± 9.9	20.9–34.9
RSV + PIV3	1/303 (0.3%)	1/61 (1.6%)	23.5 ± 0.0	23.5–23.5	36.1 ± 0.0	36.1–36.1
Total	61/303 (20.1%)	61/61 (100%)	25.4 ± 5.0	17.0–37.3	28.7 ± 5.5	15.8–38.0

Age-stratified analysis demonstrated significant differences in the frequency of viral co-infections across age groups. Co-infections were most frequent among toddlers aged 12–35 months (11.9%, 105/882), followed by infants aged 0–11 months (9.7%, 53/545) and children aged 3–5 years (7.5%, 68/902). In the remaining age groups, co-detection rates were below 4%. Notably, co-infections involving three or four viruses (24 cases) were observed predominantly among children aged 0–5 years, with two additional cases identified in children aged 7 years. The single case of quadruple viral detection occurred in a 1-year-old child. Infants aged 0–11 months had the highest rate of RSV co-detection (2.9%), followed by toddlers aged 12–35 months (2.8%), children aged 3–5 years (1.2%), adults aged ≥ 65 years (0.5%), children aged 6–17 years (0.3%), and adults aged 18–64 years (0.2%).

Among patients diagnosed with bronchitis, 104 (48.1%) had single-virus detections, while 14 (6.5%) had dual-virus detections. Among children with acute bronchiolitis, 116 (52.3%) had single-virus detections, 27 (12.2%) had dual-virus detections, and one case (0.5%) had triple-virus detection. Among patients with pneumonia, 274 (36.4%) had single-virus detections, 34 (4.5%) had dual-virus detections, and 4 (0.5%) had triple-virus detections. Only one case (0.9%) of viral co-infection was identified among patients presenting with CNS manifestations. The rate of viral co-infections among patients with bronchiolitis (12.6%, 28/222) was higher than that in the overall study population (5.7%, 297/5170) (p = 0.0001). RSV was detected as a single virus in 11.1% (24/216) of bronchitis cases, 23.0% (51/222) of bronchiolitis, 7.0% (53/753) of pneumonia, and 0.9% (1/107) of CNS. Corresponding proportions for RSV co-detection were 0.5, 7.7, 1.5, and 0%, respectively. No statistically significant difference in RSV co-infection rates was observed between inpatients (55/252) and outpatients (6/51) (p = 0.1259).

**Figure 4:**
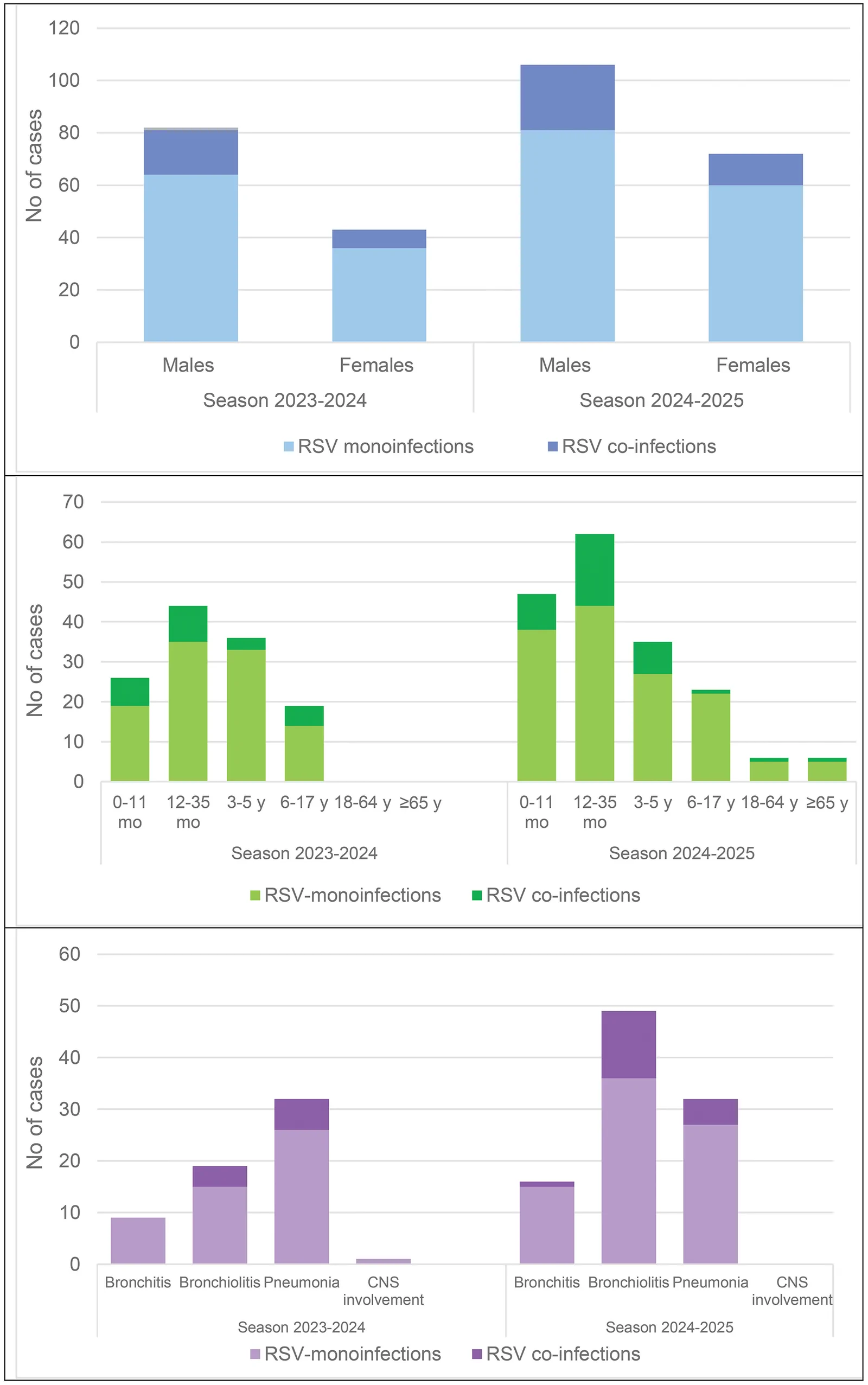
Distribution of detected RSV mono-infections and co-infections during the two seasons, categorized by sex **(A)**, age group **(B)**, and clinical diagnosis **(C)** of the patients.

**Figure 4** illustrates the number of confirmed RSV mono-infections and co-infections during the two seasons, categorized by sex (A), age group (B), and clinical diagnosis (C) of the patients. No significant differences in RSV co-infection rates by sex were observed between the two seasons: p = 0.7251 for males and p = 1.000 for females. The rates of RSV co-infections did not significantly differ across individual age groups between the two seasons. Similar conclusions can be reached about particular clinical diagnoses: bronchiolitis (p = 0.7613) and pneumonia (p = 1.000).

We compared the clinical manifestations of patients with single RSV infection with those of patients with RSV co-infection. This analysis was based on data collected at admission to the Infectious Diseases Hospital “Prof. Ivan Kirov”, Sofia, as recorded in referral forms accompanying the specimens. Among 3,001 patients with ARIs tested at this site, 69 (2.3%) were RSV-positive, including 50 (72.5%) with single-virus infection and 19 (27.5%) with RSV co-infection. Among patients with single RSV infection, the following clinical manifestations were reported: rhinorrhea in 24 (48.0%), cough in 31 (62.0%), fever in 32 (64.0%), temperature ≥38 °C in 16 (32.0%), dyspnea in 1 (2.0%), vomiting in 14 (28.0%), and diarrhea in 8 (16.0%). Among patients with RSV co-infection, the corresponding proportions were 36.8, 47.4, 52.0, 10.5, 0, 26.3, and 5.3%, respectively. No increased frequency of these clinical manifestations was observed in patients with RSV co-infection compared with those with single RSV infection.

### 3.6 Phylogenetic analysis of RSV

**Figure 5:**
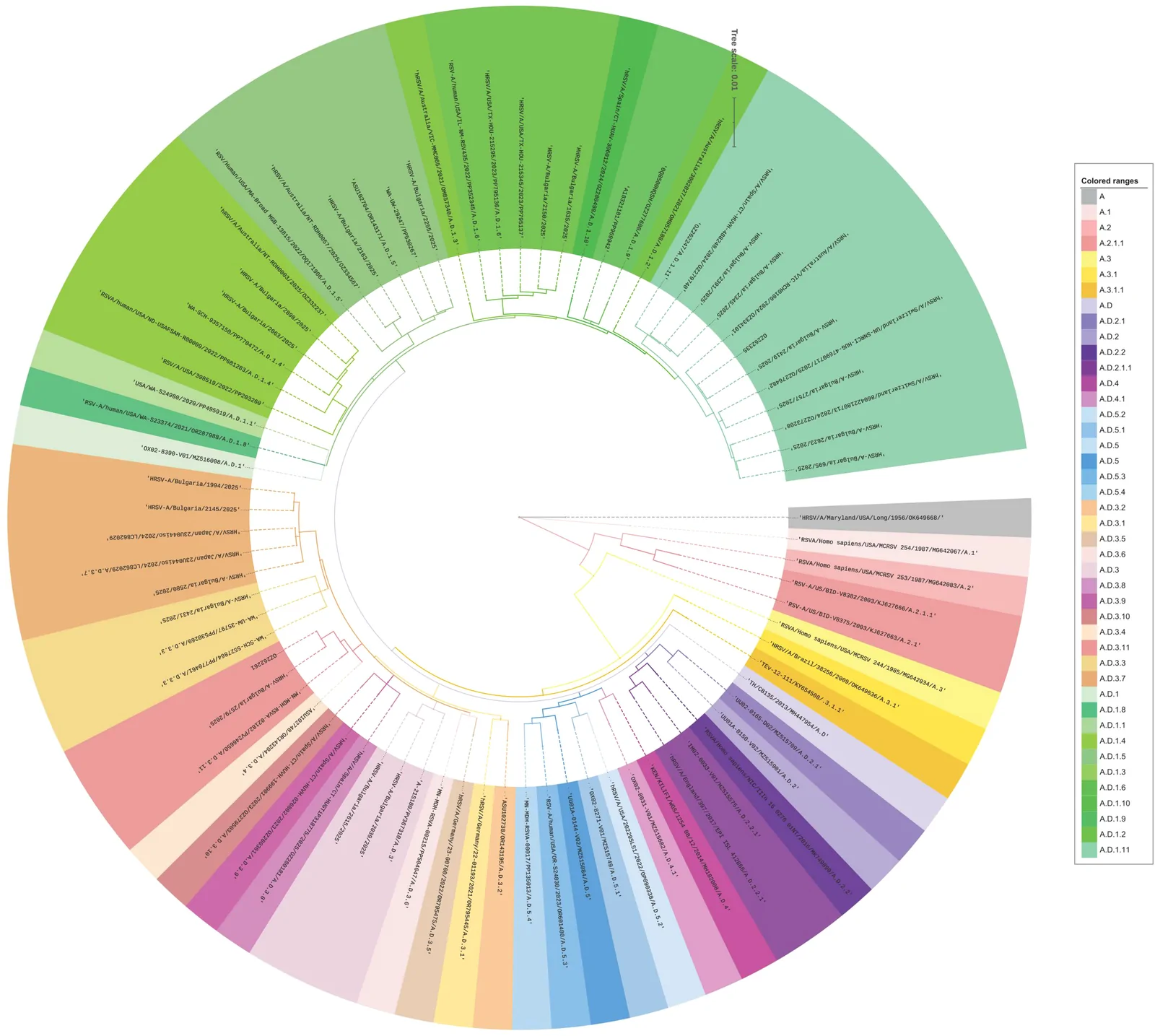
Phylogenetic analysis based on the full-length nucleotide sequences of RSV-A strains. The phylogenetic tree was constructed using the maximum-likelihood method with 1000 bootstrap iterations running within Geneious Prime software.

**Figure 6:**
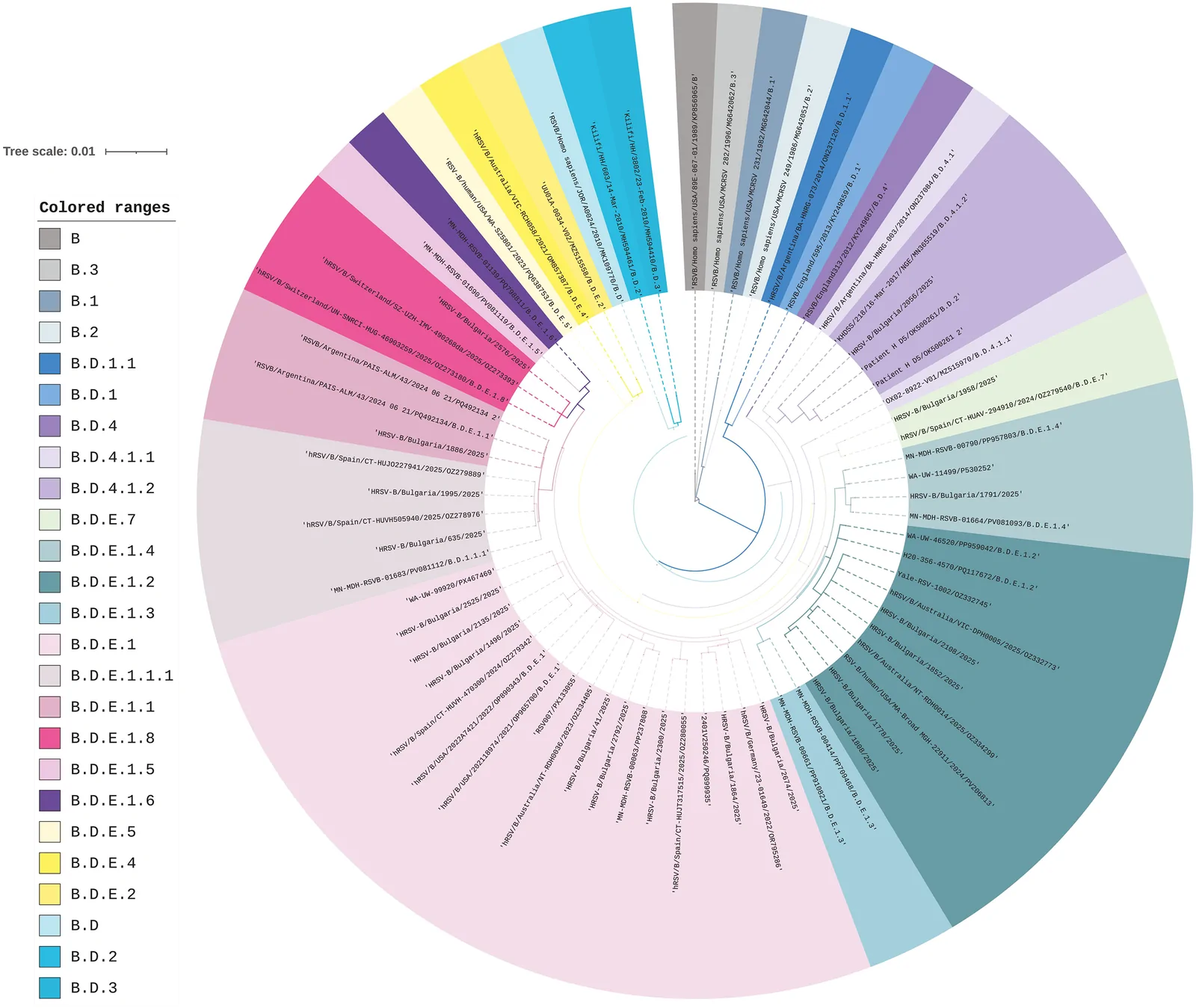
Phylogenetic analysis based on the full-length nucleotide sequences of RSV-B strains. The phylogenetic tree was constructed using the maximum-likelihood method with 1000 bootstrap iterations running within Geneious Prime software.

Phylogenetic trees based on whole-genome nucleotide sequences of RSV-A and RSV-B strains were constructed to assess the relationships between Bulgarian sequences and globally circulating strains. Phylogenetic analysis showed that all 19 RSV-A sequences belonged to G-clade GA2.3.5 and were distributed across eight lineages, all descending from lineage A.D: A.D.1.11 (n = 6), A.D.3.7 (n = 3), A.D.1.4 (n = 2), A.D.1.5 (n = 2), A.D.1.6 (n = 2), A.D.3 (n = 2), A.D.3.3 (n = 1), and A.D.3.11 (n = 1) (**Figure 5**). All 20 RSV-B sequences belonged to G-clade GB5.0.5a. Phylogenetic analysis identified seven lineages, all derived from lineage B.D: B.D.E.1 (n = 8), B.D.E.1.2 (n = 4), B.D.E.1.1 (n = 3), B.D.E.1.8 (n = 2), B.D.E.1.3 (n = 1), B.D.E.1.4 (n = 1), and B.D.E.7 (n = 1) (**Figure 6**). The predominant lineages were A.D.1.11 for RSV-A and B.D.E.1 for RSV-B. The designation “D” indicates the presence of a 72-nucleotide (RSV-A) or 60-nucleotide (RSV-B) duplication within the HVR2 of the G gene. Approximately 84% and 90% of RSV-A and RSV-B sequences, respectively, were obtained from children aged ≤ 5 years. A total of 11 sequences comprising 5 and 6 of RSV-A and RSV-B, respectively, were obtained from patients who met the definition of SARIs.

Next-generation sequencing identified three samples wherein, in addition to RSV, high-quality sequences of other respiratory viruses were detected, including SARS-CoV-2, influenza A(H1N1)pdm09, and BoV. These findings were consistent with the PCR test results.

### 3.7 Amino acid polymorphisms in major surface proteins

#### 3.7.1 Attachment (G) protein

The evolutionary dynamics of RSV are primarily driven by point mutations in the G and F genes under immune pressure. The widespread use of vaccines and mAbs may further contribute to selective pressure on circulating RSV strains. To characterize amino acid variation in the major surface glycoproteins, G and F protein sequences from 19 RSV-A and 20 RSV-B Bulgarian strains were aligned and compared with reference sequences. A total of 44 amino acid substitutions were identified in the G protein of RSV-A relative to the reference strain hRSV/A/England/397/2017. Specifically, 16 substitutions occurred in HVR1 (amino acids [aa] 67–160), one substitution in the conserved central domain (aa 161–200), and 27 substitutions in HVR2 (aa 192–321). No amino acid changes were observed in the cytoplasmic (aa 1–37) or transmembrane (aa 38–66) domains. RSV-A lineages exhibited substitutions at a median of 17 positions (range: 13–21), corresponding to 4.0–6.5% variation relative to protein length. Five substitutions (I134K, S243I, K262E, I265L, and D284G) were conserved and present in all sequences. Substitutions P71L, H90Y, L101F, and G224E were detected at high frequencies (>90%). The remaining substitutions were observed at variable frequencies across individual lineages. A comprehensive summary of amino acid variation in the G and F proteins of individual RSV-A lineages is provided in **Supplementary Table 2**. A defining feature of A.D lineages is the presence of a 72-nucleotide duplication within HVR2, resulting in duplication of a 23-amino acid motif (QKETIHSTTSEGYPSPSQVYTTS), corresponding to positions 261–283 and 285–307. Substitutions E295V, P298L/S, and Y304H occurred within this duplicated region. Analysis of N-linked glycosylation sites identified five potential sites in the RSV-A G protein at positions 85, 103, 135, 237, and 318. Two sites were within HVR1 and one within HVR2. The T320A substitution resulted in the loss of an N-linked glycosylation sequon in 12 (63.2%) strains. In HVR2, O-linked glycosylation was predicted at 35–36 serine and threonine residues, including 10 sites within the duplicated region.

Analysis of the RSV-B G protein revealed amino acid substitutions at 26 positions relative to the reference strain hRSV/B/Australia/VIC-RCH056/2019, including 10 and 16 in HVR1 and HVR2, respectively. No amino acid changes were observed in the cytoplasmic, transmembrane, or conserved domains. RSV-B lineages exhibited substitutions at a median of 11 positions (range: 9–15), corresponding to 2.9–4.8% variation relative to protein length. Substitutions A74V, T131A, I137T, I252T, and I268T were conserved across all RSV-B sequences. Substitutions S100G, P214S, P221L, and S275P were located in all lineages except B.D.E.1.3. The remaining substitutions were observed at variable frequencies across individual lineages (**Supplementary Table 3**). All B.D lineages are characterized by the presence of a 60-nucleotide duplication within HVR2, resulting in duplication of a 20-amino acid motif (TERDTSTPQSTVLDITTSKH), corresponding to positions 238–257 and 258–277. Substitutions S265P, I268T, A269V, and S275P were located within the duplicated region. Analysis of N-linked glycosylation sites identified four potential glycosylation motifs in the RSV-B G protein at positions 81, 86, 256, and 294, with two in HVR1 and two in HVR2. The K256N substitution resulted in the acquisition of an additional N-linked glycosylation sequon in 12 (60.0%) strains. The number of predicted O-linked glycosylation sites within HVR2 ranged from 37 to 40 across the analyzed sequences.

#### 3.7.2 Structural analysis of RSV-A and RSV-B G protein substitutions

**Figure 7:**
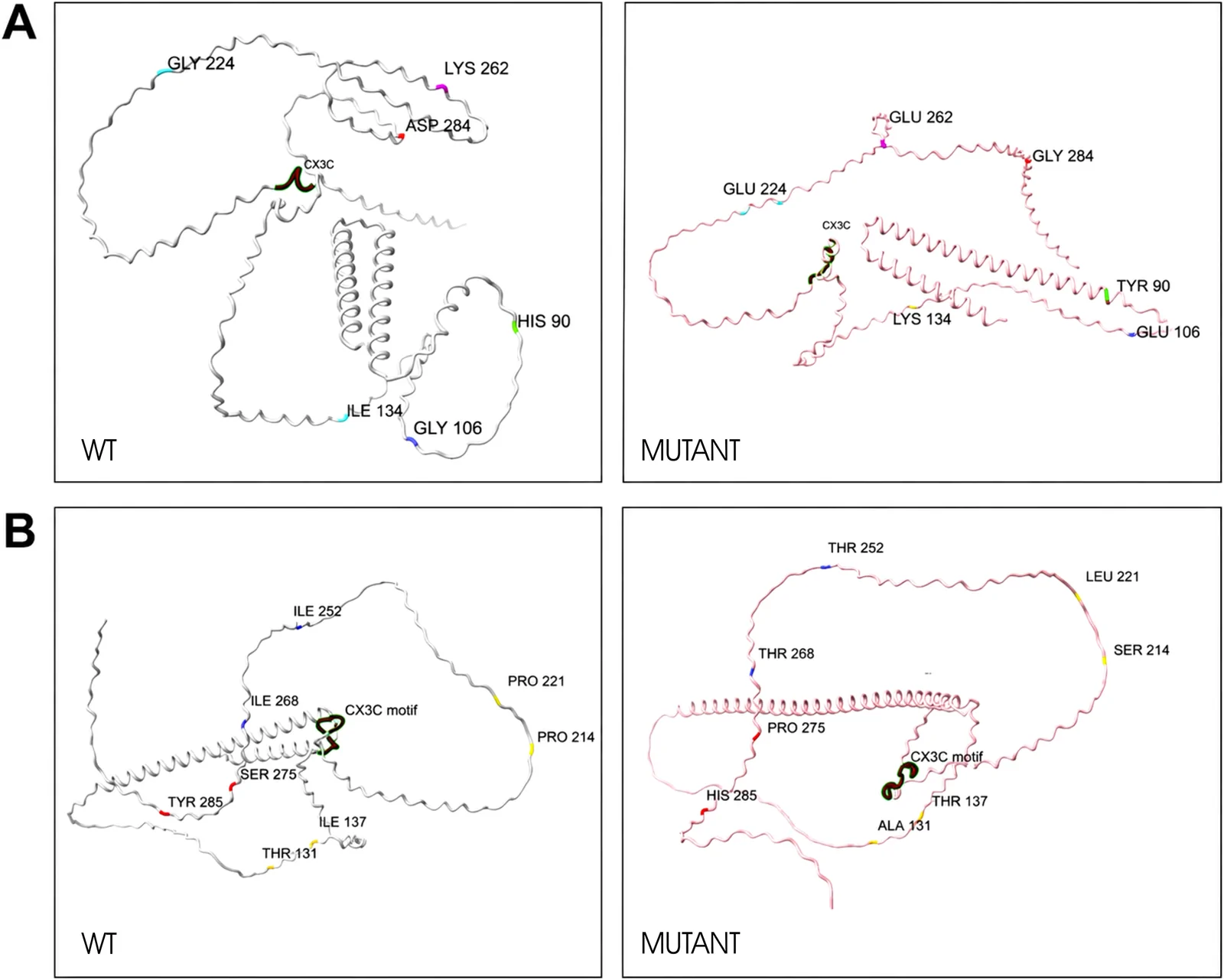
Structural localization of amino acid substitutions in RSV-A and RSV-B G glycoproteins. **(A)** Three-dimensional structural models of the wild-type (left) and mutant (right) RSV-A G glycoproteins. Selected amino acid substitutions (H90Y, G106R, I134K, G224E, K262E, and D284G) are highlighted and labeled according to their positions within the protein sequence. The conserved CX3C motif is shown in green. **(B)** Three-dimensional structural models of the wild-type (left) and mutant (right) RSV-B G glycoproteins. The substitutions T131A, I137T, P214S, P221L, I252T, I268T, S275P, and Y285H are highlighted and labeled to illustrate their spatial distribution within the protein structure. The conserved CX3C motif is shown in green. Three-dimensional protein models were generated using AlphaFold2 and visualized using UCSF ChimeraX.

Structural models generated using AlphaFold2 showed that both the reference and the studied RSV-A and RSV-B G glycoproteins retained a similar overall architecture, suggesting that the identified amino acid substitutions are unlikely to induce major conformational changes. In RSV-A, common substitutions such as H90Y, G106E, I134K, G224E, K262E, and D284G were predominantly found in exposed regions of the ectodomain in the A.D.1.11 strains studied. Similarly, in RSV-B, the common substitutions T131A, I137T, P214S, P221L, I252T, I268T, S275P, and Y285H, observed in the B.D.E.1 strains, were primarily located in the highly variable mucin-like region and were largely surface-exposed (**Figure 7**). Structural mapping revealed that the mutated residues in both subgroups were located primarily within flexible regions, whereas the conserved cysteine-rich domain containing the CX3C chemokine motif remained structurally preserved. Comparison of reference and studied models showed no significant differences in the overall fold, indicating that the observed substitutions primarily affect local physicochemical properties rather than the global structure. Several substitutions, including H90Y, I134K, K262E, T131A, P214S, P221L, and Y285H, resulted in changes in amino acid polarity or side-chain characteristics, potentially affecting antigenic determinants and antibody accessibility. Because most substitutions were located in surface-exposed regions, they may contribute to immune evasion while preserving structural integrity.

#### 3.7.3 Fusion protein

The F protein of RSV-A sequences exhibited limited amino acid variation, with substitutions identified at seven positions (overall mutation frequency: 0–0.5%) relative to the reference strain. These included three substitutions (L3S, T12I, and A23T/V) within the signal peptide (aa 1–23), three substitutions (L119H, K123R, and V127I) within the p27 region (aa 110–136), and one substitution (A518V) within the F1 subunit (aa 137–524). No amino acid substitutions were detected within the fusion peptide (aa 27–36) or the F2 subunit (aa 24–109). No amino acid changes were identified in the six major antigenic sites (Ø, I–V) of the F protein. Amino acid substitutions within the p27 region were observed in representatives of lineages A.D.1.11, A.D.3.11, and A.D.1.5. The p27 region corresponds to an internal peptide that is cleaved during maturation of the F protein into the F1 and F2 subunits (**Table 2**). Predicted N-linked glycosylation sites were identified at positions 27, 70, 116, 120, and 126 and were conserved across all RSV-A sequences.

**Table 2:** Amino acid variations identified in the F protein antigenic regions of RSV-A (n=19) and RSV-B (n=20) strains circulating in Bulgaria, 2024-2025.

Antigenic sites	Amino acid positions of antigenic sites	RSV-A F protein (aa 1-574)		RSV-B F protein (aa 1-574)	
		Amino acid changes (%)	Lineage	Amino acid changes (%)	Lineage
Ø	62–96; 195–227			S211N (19)	All except for B.D.E.1.3
				M206I (1)	B.D.E.1.3
				R209Q (1)	B.D.E.1.3
I	27–45; 312–318; 378–389			R42K (3)	B.D.E.1
				S389P (19)	All except for B.D.E.1.3
II	254-277				
III	46–54; 301–311; 345–352; 367–378				
IV	422–471				
V	55–61; 146–194; 287–300			S190 N (19)	All except for B.D.E.1.3
				R191K (1)	B.D.E.1.3
VI	422-471				
P27	110-136	L119H (4)	A.D.1.11		
		K123R (2)	A.D.3.11		
		V127I (2)	A.D.1.5		

The number of sequences harboring the indicated substitutions is shown in parentheses.

**Figure 8:**
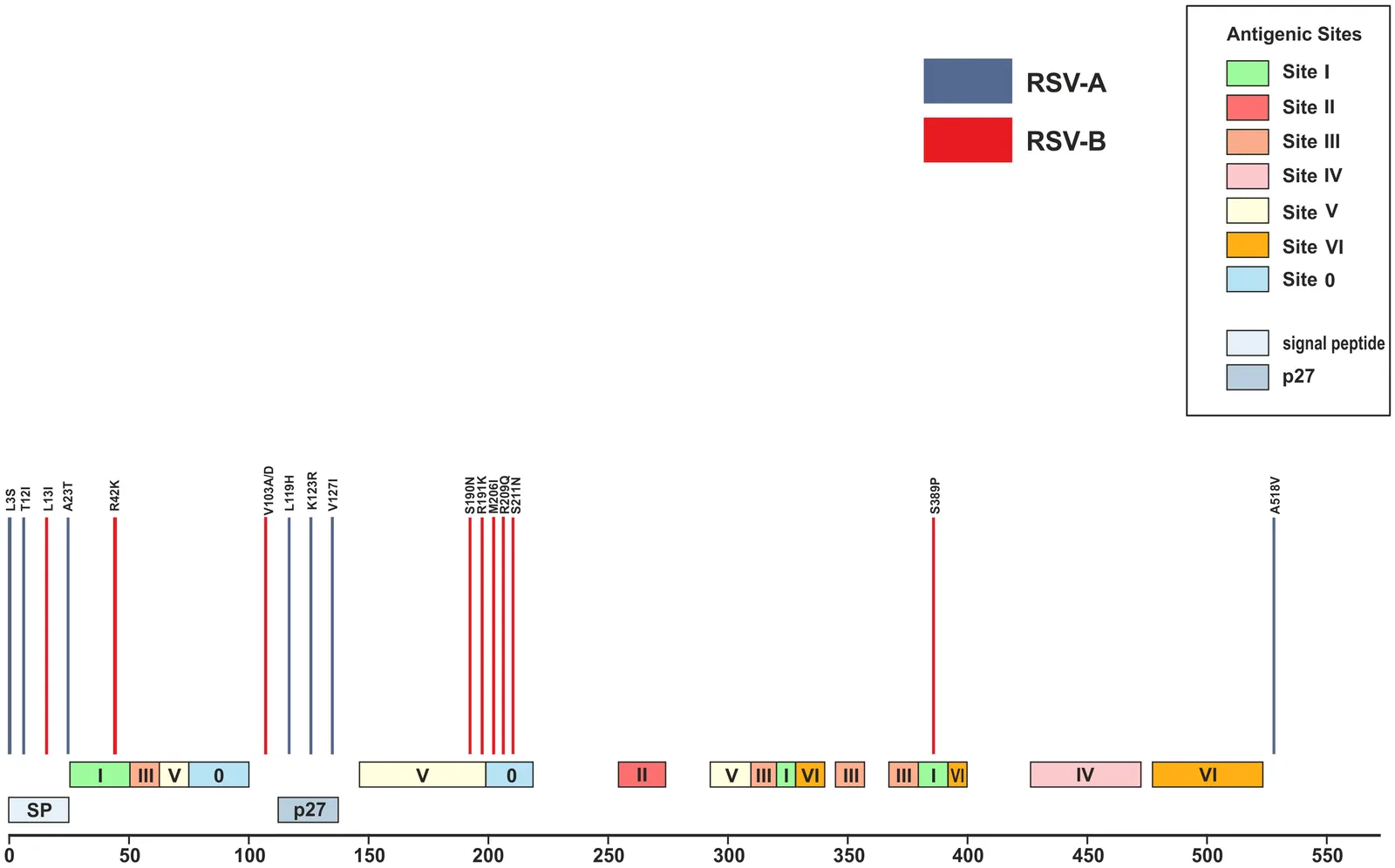
Location of amino acid substitutions identified in the RSV-A and RSV-B F proteins. Structural sites and antigenic sites Ø, I - V are shown.

The RSV-B F protein exhibited amino acid substitutions at nine positions (mutation frequency: 0.3–0.7%) relative to the reference strain. These included two substitutions (F12I and L13I) within the signal peptide, two substitutions (R42K and V103A/D) within the F2 subunit, and six substitutions (S190N, R191K, M206I, R209Q, S211N, and S389P) within the F1 subunit. No substitutions were identified within the fusion peptide. Three substitutions (S190N, S211N, and S389P) were present in all lineages except B.D.E.3. In total, seven amino acid substitutions were within three antigenic sites. The substitutions R42K and S389P were in antigenic site I, while S190N and R191K were in site V. The S211N substitution within the immunodominant Ø epitope was present in all lineages except B.D.E.1.3. The single representative of lineage B.D.E.1.3 harbored M206I and R209Q substitutions within the Ø epitope. No amino acid variations were found in the binding regions for palivizumab (site II) and clesrovimab (site IV) (**Table 2**). The remaining substitutions (F12I, L13I, and V103A/D) were outside defined antigenic regions. **Figure 8** shows the distribution of antigenic sites and associated substitutions within the F protein. Predicted N-linked glycosylation motifs at positions 27, 70, 116, 120, and 126 were conserved across all RSV-B sequences, including the reference strain. Three of these sites (N116, N120, and N126) were within the p27 region.

### 3.8 Amino acid polymorphisms in other viral proteins

The amino acid sequences of the remaining RSV-A proteins (NS1, NS2, N, P, M, SH, M2-1, M2-2, and L) were compared with those of the reference strain. Two amino acid substitutions were identified in NS1 (139 aa), two in NS2 (124 aa), three in the nucleoprotein N (391 aa), four in the phosphoprotein P (241 aa), three in the matrix protein M (256 aa), one in the small hydrophobic protein SH (65 aa), one in M2-1 (195 aa), and five in M2-2 (88 aa) (**Supplementary Table 4**). Several substitutions were conserved across all RSV-A sequences, including V352A in the N protein, L55P in the P protein, and Y24C and S44N in M2-2. The large (L) protein comprises 2,165 amino acids organized into five functional domains: RNA-dependent RNA polymerase (RdRp), capping domain (Cap), connector domain (CD), methyltransferase (MT), and carboxy-terminal domain (CTD). Amino acid substitutions observed in more than two sequences were identified at 38 positions within the L protein, with the highest number (n = 14) in the CD. The enzymatic domains RdRp, Cap, and MT contained substitutions at 12, 5, and 2 positions, respectively. Several substitutions in the L protein (P171L, R256K, Y598H, L1438Q, N1723S, and G1731D) were conserved across all sequences. The proportion of amino acid substitutions relative to protein length differed among viral proteins: NS1 (1.4%), NS2 (1.6%), N (0.8%), P (1.7%), M (1.2%), SH (1.5%), M2-1 (0.5%), M2-2 (5.7%), and L (1.8%). Although the highest absolute number of substitutions was observed in the L protein, the M2-2 protein exhibited the highest mutation frequency.

Analysis of RSV-B sequences revealed two amino acid substitutions in NS1, six in NS2, two in N, four in P, one in M, two in SH, one in M2-1, six in M2-2, and 15 in L. Only two substitutions—V97I and T1987I in N and L proteins, respectively—were conserved across all RSV-B sequences.

Lineage-defining amino acid substitutions described by the RSV RGCC were present in all study sequences.

## 4 Discussion

This study investigated the prevalence and epidemiological characteristics of RSV infections compared to those of other viral respiratory infections during the first two seasons (2023–2025) following the WHO declaration of the end of the COVID-19 public health emergency of international concern in May 2023 (WHO, 2023). Furthermore, a comprehensive genomic analysis of representative RSV strains circulating during the 2024–2025 epidemic was performed. The detection rates of RSV during the 2023–2024 and 2024–2025 seasons were quite similar, recorded at 6.6% and 6.0%, respectively. In contrast, our previous study reported a higher RSV detection rate of 13.7% during the 2022–2023 season (Korsun et al., 2021). Increased RSV activity was associated with the resurgence of seasonal respiratory viruses after the complete withdrawal of SARS-CoV-2 containment measures, a trend also observed in other countries (Munkstrup et al., 2023). Temporal and geographic variability in RSV detection rates has been reported worldwide and may depend on diverse factors, such as study population, diagnostic methods, climate, and healthcare-seeking behavior (Bimouhen et al., 2023; Kim et al., 2023).

The current study demonstrated a higher detection rate of RSV-A than of RSV-B, in contrast to our previous findings from the 2022–2023 season, which showed a predominance of RSV-B (Korsun et al., 2021). During the pre-pandemic period, year-to-year fluctuations in the predominance of RSV-A and RSV-B were observed in Bulgaria (Korsun et al., 2021), a pattern also reported in other countries (Pinana et al., 2024; Kuznetsov et al., 2025; Franco et al., 2025; Yu et al., 2025; Zhuang et al., 2025). These shifts in predominance may be influenced by herd immunity, whereby increased circulation of one RSV group is followed by the relative expansion of the other in subsequent seasons.

RSV circulation exhibits marked seasonality, with increased activity during winter and early spring (Moline et al., 2025). The temporal distribution of RSV infection in Bulgaria was consistent with patterns reported in other temperate regions of the Northern Hemisphere (Tabor et al., 2020; Kuznetsov et al., 2025). Characterizing seasonal dynamics is important for optimizing the timing of preventive interventions, including vaccination and mAb administration, as well as for anticipating periods of increased healthcare demand.

We found that RSV was detected across all age groups, with a disproportionately high concentration of cases in children under 5 years of age. This finding is consistent with global epidemiological data demonstrating increased RSV positivity in this age group (Zhuang et al., 2025; Amarin et al., 2025). In line with previous reports, RSV predominated among infants aged 0–11 months, with particularly high detection rates in those younger than 6 months, agreeing with previous reports (Yu et al., 2025). Detection rates declined with increasing age, likely reflecting the acquisition of partial immunity following repeated exposures (Madi et al., 2024a). The high RSV detection rate in infants aged < 6 months is consistent with global estimates indicating a substantial disease burden in this age group, including approximately 1.4 million hospitalizations and more than 13,000 deaths annually (Li et al., 2022). Maternal immunization with the RSV vaccine Abrysvo may protect against LRTIs during the first 6 months of life. Additionally, long-acting mAbs targeting the F protein represent an effective preventive option for infants. Although RSV is recognized as an important pathogen in older adults, contributing to hospitalization and mortality rates comparable to those of seasonal influenza (Savic et al., 2023), our study found a relatively low detection rate in individuals aged ≥ 65 years (1.6%), which is lower than estimates reported in a systematic review and meta-analysis (4.66% and 7.80% in annual and seasonal studies, respectively) (Nguyen et al., 2022). This discrepancy may reflect differences in study design, healthcare-seeking behavior, or sampling strategies. For adults aged ≥ 60 years, several RSV vaccines—including recombinant subunit vaccines (Arexvy and Abrysvo) and the mRNA-based vaccine (mResvia)—have been approved for the prevention of RSV-associated LRTIs (Yu et al., 2025).

Consistent with our previous reports, respiratory viruses were more frequently detected among inpatients than outpatients (Korsun et al., 2024). This difference was more pronounced for RSV, with detection rates of 7.3% in inpatients compared with 3.0% in outpatients. RSV was the most frequently detected virus among hospitalized children, with 81.8% of all RSV-positive cases occurring in hospitalized patients aged <5 years. These findings suggest that children younger than 5 years are disproportionately affected by RSV-associated disease requiring hospitalization. We found that RSV was the most frequently detected respiratory virus among hospitalized infants aged 0–11 months, agreeing with previous reports (Yu et al., 2025). Epidemiological studies indicate that the highest rates of RSV-associated hospitalization occur during the first months of life, including among otherwise healthy, full-term infants (Moline et al., 2024).

RSV is widely recognized as a leading cause of LRTIs in children and a major contributor to bronchiolitis and pneumonia (Hall, 2001). In our study, RSV was identified as the most common cause of bronchiolitis (30.6%) and pneumonia (12.6%) among children aged < 5 years. Both RSV-A and RSV-B were more frequently detected among inpatients than outpatients. Bronchiolitis showed the strongest association with RSV detection for both groups, whereas pneumonia was more strongly associated with RSV-A compared with RSV-B. These findings are consistent with previous observations suggesting that RSV-A may be more frequently associated with more severe LRTIs and an increased risk of hospitalization and intensive care unit (ICU) admission (Walsh et al., 1997).

In the present study, 20.1% of RSV-positive patients had co-infections with other respiratory viruses. Reported rates of RSV co-infections vary across countries, including 6.0% in the USA (Zhuang et al., 2025), 16.3% in Brazil (Bandeira et al., 2025), 17.2% in Italy (Liotti et al., 2025), 20.9% in China (Yu et al., 2025), and 34.2% in Jordan (Amarin et al., 2025). Co-detections were most frequently observed in children aged < 5 years. The frequency of clinical manifestations did not differ between patients with RSV detected as a single virus and those with RSV co-infection, suggesting no clear association between viral co-infection and disease severity. Consistent with this observation, the current literature provides inconclusive evidence regarding the impact of respiratory viral co-infections on disease severity and outcomes (Goka et al., 2014).

Phylogenetic analysis of RSV-A sequences identified eight lineages, all corresponding to ON1-derived strains. These lineages are consistent with globally circulating RSV-A lineages reported in recent studies; however, the relative prevalence of individual lineages varies geographically (Chen et al., 2025; Franco et al., 2025; Iglesias-Caballero et al., 2025; Kuznetsov et al., 2025). In our study, lineage A.D.1.11 was predominant, whereas other regions have reported different dominant lineages, including A.D.5.2 and A.D.1 in Canada (Gao et al., 2025), A.D.1.6 in the USA (Zhuang et al., 2025), A.D.5.2 in China (Wei et al., 2024) and Italy (Lai et al., 2025), and A.D.1 and A.D.5 in Portugal (Lança et al., 2025). Molecular analysis of the RSV-A G protein confirmed the presence of a 23-amino-acid duplication within HVR2, a hallmark of ON1 strains originally described in Ontario, Canada, in 2011 (Eshaghi et al., 2012). Following its emergence, the ON1 genotype rapidly became globally predominant, largely replacing previously circulating RSV-A genotypes.

RSV-B exhibited slightly lower lineage diversity than that of RSV-A. The analyzed RSV-B sequences were assigned to seven genetic lineages, all corresponding to BA-derived strains, with lineage B.D.E.1 being predominant. This lineage has also been reported as predominant in other countries, including Germany (Wetzke et al., 2025), the USA (LaVerriere et al., 2025), and China (Wei et al., 2024). The distribution of RSV-B lineages varies geographically (Chen et al., 2025; Gao et al., 2025; Iglesias-Caballero et al., 2026; Lamichhane et al., 2025; Lança et al., 2025; Kuznetsov et al., 2025). All Bulgarian RSV-B sequences contained a 20-amino acid duplication within HVR2 of the G protein, a hallmark feature of BA strains. The BA genotype was first identified in Buenos Aires, Argentina, in 1999 and subsequently became the globally predominant RSV-B genotype, replacing previously circulating RSV-B genotypes (Trento et al., 2010).

The surface glycoproteins G and F are subject to immune selection pressure and exhibit ongoing evolutionary change; accordingly, both proteins were analyzed in detail. For both RSV-A and RSV-B, the G protein showed greater amino acid variability than that of the F protein.

Structural modeling suggested that the observed substitutions do not markedly affect the overall architecture of the RSV-A and RSV-B G protein. Most substitutions were located in surface-exposed regions of the G protein, supporting their possible role in immune evasion while maintaining structural integrity. The preservation of the CX3C motif in both RSV-A and RSV-B models indicates conservation of a critical functional domain involved in CX3CR1 cellular receptor binding (Chirkova et al., 2015). These observations indicate that RSV G protein evolution is primarily driven by sequence variability within surface-exposed regions rather than by large conformational changes.

Particular attention was given to the F protein, as amino acid changes within its antigenic sites may affect the effectiveness of mAbs, similar to what has been observed for SARS-CoV-2, which has demonstrated the ability to evade all clinically approved mAbs.

In RSV-A, no amino acid substitutions were identified within the major antigenic sites (Ø, I–V), except substitutions located in the p27 region, which has been described as an additional antigenic region in the F0 precursor, although it is associated with lower neutralizing activity (Fuentes et al., 2016). The absence of substitutions in antigenic site Ø is consistent with findings from other studies (Okabe et al., 2025; Franco et al., 2025). The nirsevimab binding site (site Ø) in RSV-A remains highly conserved in recently circulating strains. Although sporadic substitutions in this region have been reported (Lamichhane et al., 2025), including variants associated with reduced susceptibility to nirsevimab (e.g., E66K and K68E) (Fu et al., 2024), such changes were not observed in the present study. Similarly, previously reported substitutions associated with reduced susceptibility to palivizumab (e.g., N262D, K272E/M/Q, and S275F/L) (Yu et al., 2025; Zhuang et al., 2025) were not detected in our sequences.

The antigenic epitopes of the RSV-B F protein showed a higher degree of variability compared with that of RSV-A. Variations at positions 206, 209, and 211 within antigenic site Ø (the target of nirsevimab) were identified. Previous studies have reported an increase in the frequency of substitutions at these positions since 2020, with S211N reaching to 100% prevalence (Wilkins et al., 2023; Piñana et al., 2024). Antigenic site Ø is located at the membrane-distal apex of the pre-fusion F trimer and is specific to the pre-fusion conformation, representing a major target of highly potent neutralizing antibodies. Substitutions in this site may indicate potential for neutralization escape and reduced effectiveness of nirsevimab. Reported reductions in neutralization include I206M (5.0-fold), Q209R (0.5-fold), and S211N (1.2-fold), whereas K68N has been associated with a substantially greater reduction (29.9-fold) (Wilkins et al., 2023). Resistance-associated substitutions have been described in both clinical trials and surveillance studies. In clinical trials, substitutions such as I64T, K68E/N, and N208S/D have been detected in patients receiving nirsevimab (Ahani et al., 2023). In addition, breakthrough infections involving RSV-B have been associated with substitutions such as N208D (the dominant resistance-associated substitution) and combinations including I64M and K65R (considered a minority resistance-associated substitution) (Fourati et al., 2025). Importantly, none of these well-characterized resistance-associated substitutions were identified in the sequences analyzed in the present study. These findings support the continued susceptibility of circulating RSV-B strains to currently approved mAbs. Nevertheless, the emergence and increasing frequency of substitutions within antigenic site Ø underscore the importance of ongoing molecular surveillance of RSV.

In our study, two amino acid substitutions were identified in antigenic site I and two in antigenic site V, which is the target of mAbs suptavumab. Studies have reported an increase in the frequency of substitutions such as S190N and S389P since 2020 (Chen et al., 2025). Both the study sequences and the reference sequence harbored L172Q/S173L substitutions within antigenic site V. The global predominance of these substitutions has been associated with reduced neutralization by suptavumab and was linked to the failure of phase 3 clinical trials of this mAb (Simões et al., 2021). In contrast, no amino acid substitutions were identified in antigenic sites II (target of palivizumab) or IV (target of clesrovimab). Studies have shown that antigenic sites III and IV exhibit minimal variability, site II shows limited variability, and sites V and Ø are more variable (Okabe et al., 2025).

The surface glycoproteins G and F are extensively glycosylated through N-linked and O-linked glycans, which can hinder antibody access to antigenic epitopes and contribute to immune evasion (Holmes, 2013). Changes in glycosylation patterns, including the gain or loss of glycosylation sites, may influence viral antigenicity and virulence (Madi et al., 2024b). Together, amino acid substitutions and glycosylation of the G and F proteins represent key mechanisms driving RSV evolution, with potential implications for viral fitness, transmissibility, and immune escape.

Full-length sequencing of the remaining viral proteins revealed a relatively low number of amino acid substitutions compared with those of reference sequences. The M2-2 protein, which regulates the balance between transcription and replication, exhibited higher relative variability, consistent with previous reports (Bender et al., 2024). Amino acid substitutions were also identified in conserved enzymatic regions of the L protein, specifically within the RdRp, Cap, and MT domains (Cao et al., 2024). Such substitutions may have implications for antiviral susceptibility, particularly for agents targeting polymerase activity, such as the nucleoside analogue ribavirin (Liuzzi et al., 2005). In addition, the higher number of substitutions observed in the connector domain (CD) may be relevant to the activity of emerging inhibitors targeting this region (Bonneux et al., 2024).

This study has several limitations. The study population was predominantly composed of children and adolescents (< 18 years), with limited representation of adults aged 18–64 years and ≥ 65 years. Future studies should aim to include a more balanced age distribution. In addition, the relatively small number of sequences within individual genetic lineages limited our ability to assess age-specific and temporal patterns in lineage distribution. Expanded WGS efforts are needed to provide a more comprehensive understanding of RSV genetic diversity at the national level. Furthermore, although multiple amino acid substitutions were identified, their functional impact on disease severity and the effectiveness of preventive interventions was not assessed. Detailed clinical severity parameters (e.g., oxygen requirement, ICU admission, and duration of hospitalization) were not systematically available, which limited the assessment of disease severity. Nonetheless, despite these limitations, this study provides a comprehensive overview of circulation patterns and a detailed genomic characterization of RSV during the first two post-pandemic seasons.

## 5 Conclusion

This study demonstrates substantial circulation of respiratory viruses, with RSV representing a leading cause of severe respiratory illnesses in young children. The co-circulation of multiple RSV lineages was observed, underscoring the importance of sustained molecular surveillance. Importantly, resistance-associated substitutions in the F protein were not detected within regions targeted by currently approved mAbs, supporting the continued effectiveness of these preventive strategies. Therefore, ongoing monitoring of genetic variation in the F gene remains essential for the early detection of emerging resistance-associated variants under selective pressure.

## Data Availability

The datasets presented in this study can be found in online repositories. The names of the repository/repositories and accession number(s) can be found in the article/**Supplementary Material**.
