# Correction: Prevalence and genetic diversity of respiratory syncytial viruses circulating in Bulgaria, 2023-2025

**DOI:** 10.3389/fcimb.2026.2007703

**Published:** 2026-10-05

**Authors:** Neli Korsun, Ivelina Trifonova, Yordanka Uzunova, Petar Velikov, Diyana Pavlova, Iliana Grigorova, Ivan Ivanov, Daniel Ivanov, Silviya Voleva, Iva Christova

**Affiliations:** 1 National Reference Laboratory “Influenza and ARD”, Department of Virology, National Center of Infectious and Parasitic Diseases, Sofia, Bulgaria; 2 Center of Competence “ImmunoPathogen”, Sofia, Bulgaria; 3 University Hospital “Lozenetz”, Sofia, Bulgaria; 4 Department for Infectious Diseases, Parasitology and Tropical Medicine, Medical University of Sofia, Sofia, Bulgaria; 5 Infectious Disease Hospital “Prof. Ivan Kirov”, Sofia, Bulgaria

**Keywords:** respiratory syncytial virus, acute respiratory infections, genetic diversity, monoclonal antibodies, whole-genome sequencing, three-dimensional protein model

An incorrect number was provided for the Ministry of Education and Science, Bulgaria. The correct number is “KP‐06‐H73/7‐05.12.2023 and КP-06-Н93/4-09.12.2025”. The correct **Funding** statement is:

“The author(s) declared that financial support was received for this work and/or its publication. This study was supported by grants from the Ministry of Education and Science, Bulgaria (Scientific Research Fund, contracts KP‐06‐H73/7‐05.12.2023 and КP-06-Н93/4-09.12.2025), and by the Program “Research, Innovation and Digitalization for Smart Transformation” (PRIDST) 2021-2027, Procedure BG16RFPR002-1.014 “Sustainable Development of Centers of Excellence and Centers of Competence including specific infrastructures or their consortia from the National Roadmap for Research Infrastructure (NRRI)”.”

The original version of this article has been updated.

