# Supplementary material for: Prevalence and genetic diversity of respiratory syncytial viruses circulating in Bulgaria, 2023-2025
Source: Front Cell Infect Microbiol. 2026 Jul 24;16:1844212. doi: 10.3389/fcimb.2026.1844212 (PMC13447448; doi:10.3389/fcimb.2026.1844212)
Supplement: Supplementary file 1 [file DataSheet1.pdf]

Supplementary Appendix

All genome sequences and associated metadata supporting the findings of this study can be accessed through the persistent digital object identifier https://doi.org/10.55876/gis8.260330by

In addition to the minted DOI, GISAID also communicates the aggregation of GISAID accession numbers (EPI_ISL_IDs) through the corresponding EPI_SET_260330by identifier to facilitate both, the acknowledgment of all data contributors and the direct retrieval of the underlying data from GISAID used in this study.

RSV Data Summary

GISAID Identifier	Digital Object Identifier	Number of individual viruses	Data Collection range	Number of countries/territories
EPI_SET_260330by	https://doi.org/10.55876/gis8.260330by	39	2025-01-06 to 2025-04-11	1
