# Supplementary material for: Prevalence and genetic diversity of respiratory syncytial viruses circulating in Bulgaria, 2023-2025
Source: Front Cell Infect Microbiol. 2026 Jul 24;16:1844212. doi: 10.3389/fcimb.2026.1844212 (PMC13447448; doi:10.3389/fcimb.2026.1844212)
Supplement: Supplementary file 2 [file DataSheet2.pdf]

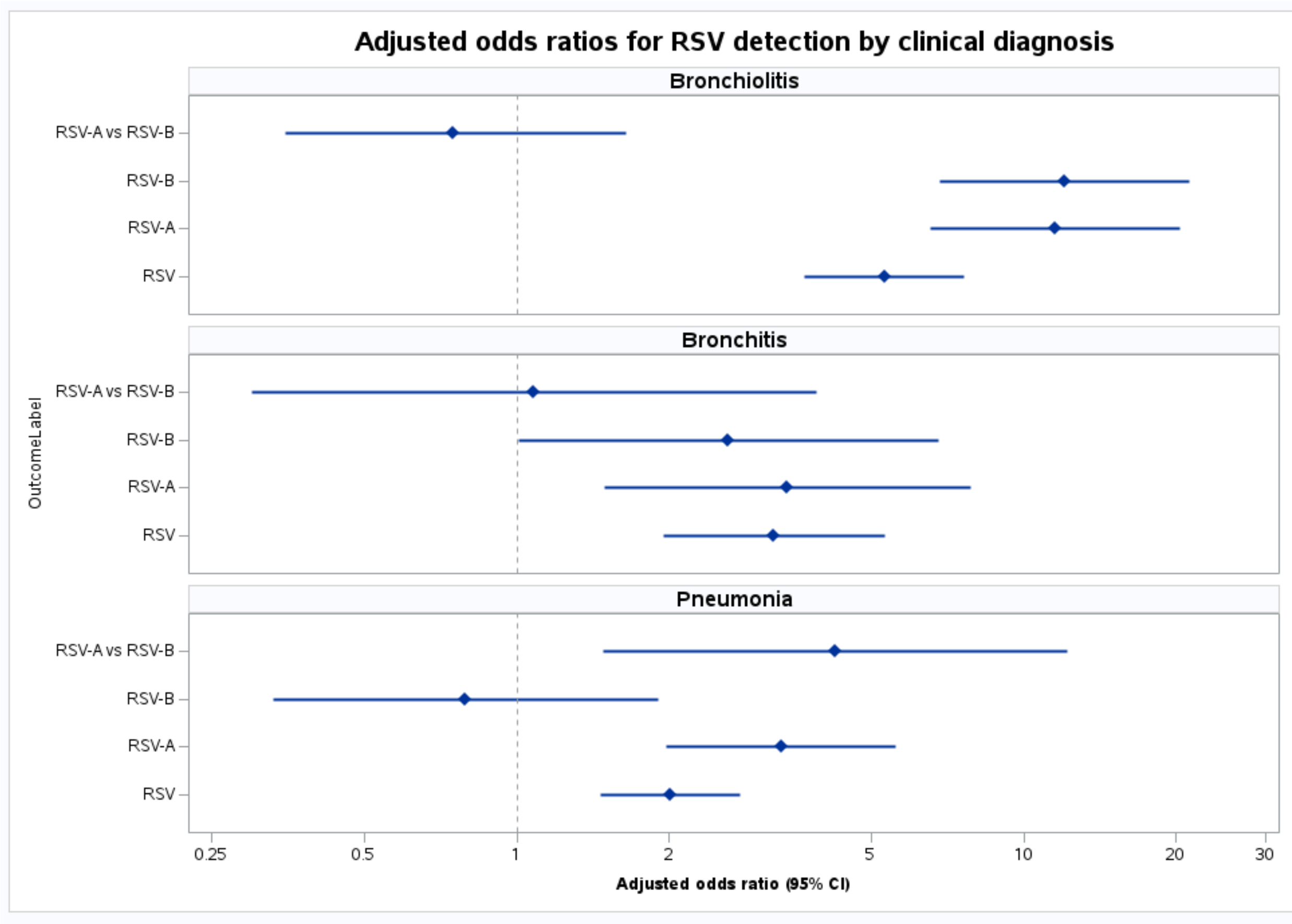

Supplementary Figure. Adjusted odds ratios for RSV detection by clinical diagnosis. Forest plot showing adjusted odds ratios and 95% confidence intervals for RSV, RSV-A, RSV-B, and direct comparison of RSV-A versus RSV-B according to clinical diagnosis, with upper respiratory tract infection (URTI) as the reference category. Adjusted models include age and sex.
