# Supplementary material for: Prevalence and genetic diversity of respiratory syncytial viruses circulating in Bulgaria, 2023-2025
Source: Front Cell Infect Microbiol. 2026 Jul 24;16:1844212. doi: 10.3389/fcimb.2026.1844212 (PMC13447448; doi:10.3389/fcimb.2026.1844212)
Supplement: Supplementary file 3 [file DataSheet3.pdf]

Supplementary Table 1. Univariable and multivariable logistic regression analysis of factors associated with RSV detection

Variable	Category	Univariable OR (95% CI)	p-value	Multivariable aOR (95% CI)	p-value
**Sex**	Female	Reference	-	Reference	-
	Male	1.55 (1.22-1.98)	0.0004	1.41 (1.10-1.82)	0.0074
**Age group**	18-64 years	Reference	-	Reference	-
	0-11 months	11.36 (4.88-26.45)	<0.0001	6.64 (2.80-15.71)	<0.0001
	12-35 months	10.01 (4.36-23.01)	<0.0001	6.22 (2.67-14.47)	<0.0001
	3-5 years	6.47 (2.79-15.01)	<0.0001	4.91 (2.10-11.47)	0.0002
	6-17 years	1.72 (0.73-4.09)	0.2162	1.46 (0.61-3.48)	0.3919
	≥65 years	1.15 (0.37-3.58)	0.8155	1.05 (0.33-3.30)	0.9363
**Clinical diagnosis**	URTI	Reference	-	Reference	-
	Bronchiolitis	10.82 (7.75-15.11)	<0.0001	5.31 (3.69-7.63)	<0.0001
	Bronchitis	3.13 (1.94-5.04)	<0.0001	3.21 (1.95-5.31)	<0.0001
	Pneumonia	2.23 (1.64-3.04)	<0.0001	2.01 (1.46-2.75)	<0.0001
	CNS involvement	0.40 (0.10-1.62)	0.1979	0.55 (0.13-2.26)	0.403

Abbreviations: RSV, respiratory syncytial virus; OR, odds ratio; aOR, adjusted odds ratio; CI, confidence interval; URTI, upper respiratory tract infection; CNS, central nervous system.

Note: The multivariable model included sex, age group, and clinical diagnosis. Female sex, adults aged 18-64 years, and URTI (133 / 3391 RSV-positive cases) were used as reference categories. Records with missing or unspecified diagnoses were excluded from the logistic regression model.
