# Supplementary material for: Prevalence and genetic diversity of respiratory syncytial viruses circulating in Bulgaria, 2023-2025
Source: Front Cell Infect Microbiol. 2026 Jul 24;16:1844212. doi: 10.3389/fcimb.2026.1844212 (PMC13447448; doi:10.3389/fcimb.2026.1844212)
Supplement: Supplementary file 4 [file DataSheet4.pdf]

Supplementary Table 2. Amino acid substitutions in RSV-A G and F proteins detected in individual genetic lineages

Substitution	A.D.1.11 (n=6)	A.D.3.7 (n=3)	A.D.1.4 (n=2)	A.D.1.5 (n=2)	A.D.1.6 (n=2)	A.D.3 (n=2)	A.D.3.3 (n=1)	A.D.3.11 (n=1)
**G protein (321 aa)**								
H67N		3						
P71L	6	3	2	2	2	2	1	
H90Y	6	3	2	2	2	2	1	
L101F	6	3	2	2	2	2	1	
G106R/E		3 (R)			1 (E)			
T113I		3				2	1	1
P120L					2	2		
S121I					2			
A122V			2					
V131D		3				2	1	1
I133V			2					
I134K	6	3	2	2	2	2	1	1
L142S	6		2	2	2			
P143S					2			
S144I			2					
R151Q		3						
N178G		3				2	1	1
K204N							1	
P217S					2			
K221R		3						
G224E/V/A	6 (E)	3 (E)	2 (V)	1 (A)	2 (E)	2 (E)	1 (E)	1 (E)
V225A	6		2					
L226F			2					
N242T					2			
S243I	6	3	2	2	2	2	1	1
T245A						2		1
L247P						2		
L248P						2		
H258Q		3				2	1	1
S260N						2		
K262E	6	3	2	2	2	2	1	1
I265L	6	3	2	2	2	2	1	1
H266L		3				2	1	1

S270P			2					
Y273H			2					
P274L		3						
P276L	6							1
D284G	6	3	2	2	2	2	1	1
E295V	6							
P298L/S		3 (L)	2 (S)			2	1	1
Y304H	5		2	2	2			
L314P	6		2					
T319K/I/A		3 (K)		2 (I)	2 (A)			
T320A	6		2	2	2			
Mutation frequency*	5%	6.5%	6.5%	4%	5.9%	6.2%	5%	4.4%
**F protein (574 aa)**								
L3S			2					
T12I		3				2	1	1
A23T						2	1 (V)	1
L119H	4							
K123R								1
V127I				2				
A518V	6							
Mutation frequency*	0.3%	0.2%	0.2%	0.2%	0	0.3%	0.3%	0.5%

*The mutation frequency was calculated as the number of positions with an amino acid change relative to the total length of the G protein (n/321) and F protein (n/574).

Supplementary Table 3. Amino acid substitutions in RSV-B G and F proteins detected in individual genetic lineages

Substitution	B.D.E.1 (n=8)	B.D.E.1.2 (n=4)	B.D.E.1.1 (n=3)	B.D.E.1.8 (n=2)	B.D.E.1.3 (n=1)	B.D.E.1.4 (n=1)	B.D.E.7 (n=1)
**G protein (310 aa)**							
A74V	8	4	3	2	1	1	1
L91F					1		
R98K							1
S100G	8	4	3	2		1	1
A107T						1	
H112Y				2			
A116T		4					

T131A	8	4	3	2	1	1	1
I137T	8	4	3	2	1	1	1
P150S							1
P214S	8	4	3	2		1	1
L217P					1		
P221L	8	4	3	2		1	1
V249A				2			
I252T	8	4	3	2	1	1	1
T254I		4					
K256N	8		3	2			
S265P	2			1			
I268T	8	4	3	2	1	1	1
A269V	2	4	1				
S275P	8	4	2	2		1	1
Y285H	8	4	3	2		1	
P289L					1		
A301I					1		
E303G		4					
S307P		4					
Mutation frequency*	4.2%	4.8%	3.9%	4.5%	2.9%	3.5%	3.5%
**F protein (574 aa)**							
F12I		4					
R42K	3						
S190N	8	4	3	2		1	1
R191K					1		
M206I					1		
R209Q					1		
S211N	8	4	3	2		1	1
P311H					1		
S389P	8	4	3	2		1	
Mutation frequency*	0.7%	0.7%	0.5%	0.5%	0.7%	0.5%	0.3%

*The mutation frequency was calculated as the number of positions with an amino acid change relative to the total length of the G protein (n/310) and F protein (n/574).

Supplementary Table 4. Amino acid substitutions in NS1, NS2, N, P, M, SH, M2-1, M2-2, and L proteins in RSV-A and RSV-B. The number of sequences harboring indicated substitutions is shown in parentheses.

Viruses	NS-1	NS-2	N	P	M	SH	M2-1	M2-2	L
RSV-A	G2D (3)<br>E91D (2)	T28A (2)<br>R50K (2)	I65V (2)<br>K84R (1)<br>V352A (19)	P34S (2)<br>L55P (19)<br>I62T (2)<br>T92M (8)	K25R (6)<br>M43I (12)<br>I159T (2)	I38S (8)	T180A (2)	Y24C (19)<br>S44N (19)<br>H45Y (1)<br>D62G (2)<br>T77A (8)	Substitutions detected in 2 or more strains at 38 positions
RSV-B	K74E (3)<br>I93M (1)	T3A (1)<br>D6N (4)<br>Y28I (1)<br>K80R (1)<br>T82A (1)<br>E83K (3)	T120A (1)<br>V97I (20)	S67G (1)<br>P73S (1)<br>D225N (3)<br>D229G (1)	T89I (1)	I49M (2)<br>D64N (2)	I125V (1)	S24G (18)<br>M27T (19)<br>D35N (18)<br>Q38K (1)<br>C49F (17)<br>C49Y (1)	Substitutions detected in 2 or more strains at 15 positions
